# m^6^A Reader HNRNPC Facilitates Adipogenesis by Regulating Cytoskeletal Remodeling through Enhanced *Lcp1* mRNA Stability

**DOI:** 10.14336/AD.2024.0132

**Published:** 2024-05-14

**Authors:** Wenhua Xie, Yewei Cui, Lingzhi Yue, Ting Zhang, Chenglong Huang, Xinyu Yu, Dan Ma, Dongfang Liu, Rui Cheng, Xueya Zhao, Xi Li

**Affiliations:** ^1^Institute of Life Sciences, School of Basic Medicine, Chongqing Medical University, Chongqing, China.; ^2^Center of Obesity and Metabolic Diseases, Department of General Surgery, The Third People’s Hospital of Chengdu, Affiliated Hospital of Southwest Jiaotong University & The Second Affiliated Hospital of Chengdu, Chongqing Medical University, Chengdu, China.; ^3^Department of Clinical Laboratory, University-Town Hospital of Chongqing Medical University, Chongqing, China.; ^4^The second Affiliated Hospital, Chongqing Medical University, Chongqing, China.

**Keywords:** Aging, m^6^A, HNRNPC, Adipogenesis, LCP1, Cytoskeletal

## Abstract

Reduced adipogenesis is a prominent characteristic of aging adipose tissue and is closely tied to the development of metabolic disorders associated with aging. Epigenetic modification plays a crucial role in the aging process, yet the role of N^6^-methyladenosine (m^6^A), the most prevalent RNA modification, in regulating adipose tissue aging remains uncertain. Our study found that levels of m^6^A and its recognition protein, heterogeneous nuclear ribonucleoprotein C (HNRNPC), decrease in adipose tissue as individuals age. Lower levels of HNRNPC were also linked to reduced adipogenesis during aging. Through loss and gain of function experiments with HNRNPC, we established a positive correlation between HNRNPC and adipogenesis in vitro. *Hnrnpc-APKO* mice displayed decreased adipogenesis, increased insulin resistance, elevated expression of aging-related and inflammation-related genes, decreased lipogenesis-related genes, and other metabolic disorders compared to their littermates. Additionally, we discovered that HNRNPC facilitated the stability of lymphocyte cytosolic protein 1 (*Lcp1)* mRNA by binding to the m^6^A motif of LCP1. Overexpression of LCP1 mitigated the inhibition of adipogenesis caused by decreased HNRNPC through modulation of cytoskeletal remodeling. Finally, our findings demonstrate that anti-aging treatments could enhance HNRNPC levels. In conclusion, HNRNPC is positively associated with reduced adipogenesis during aging, and increacing HNRNPC levels through anti-aging treatments highlights its potential as a therapeutic target for addressing metabolic imbalances in adipose tissue related to aging.

## INTRODUCTION

The World Health Organization (WHO) predicts that the global population of adults over the age of 60 will reach 2.1 billion by 2050. As individuals age, there is a gradual decline in tissue performance which increases the risk of many diseases, such as type 2 diabetes, cardiovascular diseases, and cancers [1, 2]. Many metabolic diseases are linked to aberrant adipose tissue function [3, 4]. Single-cell sequencing analysis of multiple tissues in aging mice and flies indicates the most substantial quantitative changes in adipose tissue, which undergoes senescence first compared to other tissues [2, 5, 6]. Furthermore, several studies indicate that adipose tissue aging can lead to ectopic lipid storage, adipokines dysregulation, insulin resistance, and other metabolic disorders [7, 8].

Aged adipose tissue is associated with an increase in fibrosis and a decrease in the ability of adipogenesis [3, 7, 9]. Adipogenesis is the process by which stem cells differentiate into mature adipocytes, which mitigates ectopic fat deposition and improves insulin sensitivity [10, 11]. As a result, it is critical to investigate new mechanisms that can regulate adipogenesis during aging.

Epigenetic modification profiles have been demonstrated to undergo alterations with aging [12, 13]. m^6^A is an RNA-associated epigenetic modification that is involved in a variety of biological processes and functions through the collaboration of methyltransferases (METTL3, METTL14, etc), demethyltransferases (FTO and ALKBH5), and readers (YTHDF1, YTHDF2, etc). The readers recognize methylated targets, cause target degradation and splicing, and participate in several physiological or pathological processes [14-16].

Some studies have found that m^6^A regulators are involved in aging, such as METTL3 promotes fibroblast-like synoviocytes senescence by regulating the autophagy-GATA4 axis [17]. Furthermore, ALKBH5 facilitates *Cyp1b1* mRNA degradation to alleviate mesenchymal stem cells (MSC) senescence [18]. These m^6^A regulators have also been reported to modulate the process of adipogenesis. METTL3 is a positive regulator of adipogenesis in 3T3-L1 [19], while ALKBH5 is a negative regulator of adipogenesis in MSC [20]. However, the underlying mechanism of m^6^A regulators in adipose tissue aging, especially aging associated adipogenesis decay remains unclear.

HNRNPC is widely distributed in multiple tissues and is an important regulator of various biological processes [21-23]. Previsous studies have demonstrated the potential role of HNRNPC in lipid-related metabolism. Particularly, HNRNPC expression has been closely correlated with BMI [24]. In gastric cancer peritoneal metastasis, the interaction between LINC00924 and HNRNPC has been shown to promote fatty acid oxidation and uptake, thereby regulating lipid metabolism reprogramming [25]. Additionally, in chronic lymphocytic leukemia, m^6^A circTET2-modified cyclic RNA interacts with HNRNPC, influencing fatty acid oxidation and mTORC1 signaling pathways, ultimately promoting cell proliferation [26]. While adipose tissue is known to be the largest energy reservoir in the body and plays a crucial role in regulating lipid metabolism and energy homeostasis, the potential involvement of HNRNPC in adipose tissue or its specific role in this context remains unclear.

In our study, we found a reduction in HNRNPC expression, the most significant difference among the m^6^A regulators in human and mouse aging adipose tissue and cells. HNRNPC depletion inhibited adipogenesis *in vitro* and *in vivo*, and *Hnrnpc-APKO* mice exhibited metabolic disorders such as insulin resistance, reduced energy expenditure, and increased mRNA expression of aging genes. In addition, we found that HNRNPC enhanced the stability of *Lcp1* and subsequently improved adipogenesis via cytoskeleton repair.

## MATERIALS AND METHODS

### Human studies

Subcutaneous adipose tissue sections of human subjects were obtained from individuals who underwent laparoscopic surgery at Chengdu No. 3 Hospital. Clinical information about the human donors is shown in Table 1.

**Table 1:** The clinical information of human donors

	Gender	Age
**Donor1**	Male	51
**Donor2**	Male	58
**Donor3**	Male	58
**Donor4**	Female	67
**Donor5**	Male	70
**Donor6**	Male	64

### Animals

Male of Young (6 months old) and old mice (21 months old) (C57BL/6J background) were obtained from Chongqing Medical University’s Animal Experiment Center. *Hnrnpc^pdgfra/+^* (APKO) mice were constructed using the CRISPR-Cas9 technique at Saiye Biotechnology. Mice were housed and kept in 12-hour light/dark cycles with free access to water and food. At 8 weeks, the male *Hnrnpc^flox/+^* (Flox) mice and *Hnrnpc^pdgfra/+^* (APKO) mice started the experiment and were fed a standard diet (HuaFukang, China) until 13 weeks. Weekly body weight measurements were taken. The mice's core body temperature was monitored using an anal thermometer before they were sacrificed, and the blood and tissue were harvested.

### Energy metabolism measurement

Energy metabolism experiments were performed using the CLAMS Metabolic Measurement System (Columbus Instruments, USA). Male APKO and Flox mice were fed standard chow diet until they reached 10 weeks of age. Subsequently, they were individually housed in metabolic cages to facilitate ad libitum access to food and water. Oxygen consumption, carbon dioxide production, and heat production have been measured in these mice.

### Glucose tolerance test and insulin sensitivity test

To determine systemic glucose homeostasis and insulin sensitivity in male APKO and Flox mice, we performed glucose tolerance test (GTT) in mice fed normal chow for up at 8 weeks and insulin tolerance test (ITT) at week 9. After fasting for 14 to 16 h, blood glucose concentrations were measured using a blood glucose meter, and GTT was performed after an intraperitoneal injection of 10% glucose solution (2 g/kg body weight) into the mice. Blood glucose levels were checked at 0, 30, 60, 90, and 120 min after the glucose injection. After fasting the mice for 6 h, ITT was performed with an intraperitoneal injection of human insulin (0.75 U/kg body weight). Blood glucose levels were checked at 0, 30, 60, 90, and 120 min after insulin injection.

### Blood biochemical analysis

Serum levels of triglycerides (TG) and non-esterified fatty acids (NEFA) were measured using assay kits (Nanjing Jiancheng Bioengineering). Briefly, for the TG, we set the blank wells, calibration wells, and sample wells. Then, we mixed 2.5 µL of the corresponding liquid with 250 µL of the working solution and incubated the mixture at 37 °C for 10 min. The wavelength 500 nm enzyme labeling instrument was used to determine the absorbance value of each hole. For the NEFA, we set the blank wells, calibration wells, and sample wells. Then, we added 4 µL of the corresponding liquid to 200 µL of the working solution and mixed well before incubating the solution at 37 °C for 5 min. For determining absorbance value A1 (main wavelength 546 nm, auxiliary wavelength 600 nm), we added 50 µL of reagent 2, mixed well, and incubated for 5 min. Then, we read the absorbance value A2 (main wavelength 546 nm, auxiliary wavelength 600 nm) and calculated A2-A1.

### H&E staining and immunohistochemistry

For histology studies, we stained 5-μm thick subcutaneous and epididymal adipose tissues from the mice with hematoxylin and eosin (H&E). We then photographed it. Immunohistochemistry of adipose tissue sections from human was performed after deparaffinization and rehydration. The adjacent 5-µm thick fat sections were incubated with 10 mmol/L citric acid (pH 6.0) and heated in a microwave (2 min at 700 W, repeated twice) to recover antigenicity. The tissue sections were incubated in a solution of 3% H_2_O_2_ to quench the peroxidase for 15 min. Nonspecific binding was blocked using 10% normal goat serum (ZSGB-bio, pv-6000). The adipose tussue section was incubated with an HNRNPC antibody (Invitrogen, MA1-24631, 1:400), and the staining images were captured and digitalized by a fluorescence microscope (Olympus, Japan).

### Cell culture and white adipocyte differentiation

The 3T3-L1 cells were cultured with Dulbecco’s modified Eagle’s medium (DMEM) with 10% Bovine Calf Serum (Sigma, USA), 1% Biotin (Sigma, USA), and 1% penicillin and streptomycin (Beyotime Biotechnology, China) in 5% CO_2_. Two days post confluence, we exposed the cells to DMEM with 10% Fetal Bovine Serum (ThermoFisher, USA), 1% Biotin, and 1% penicillin and streptomycin containing 0.5 mM 3-isobutyl-1-methyl-xanthine (Sigma, USA), 1 µM dexamethasone (Sigma, USA), and 1µg/mL insulin (Solarbio, China). After two days exposure in above medium, the cells were maintained in a medium with 1µg/mL insulin (Solarbio, China). After induction into mature adipocytes, cell harvesting was performed for western blot. For the RIP-RT-qPCR, the 3T3-L1 cells were harvested before confluence.

The SVF was obtained from adipose tissue isolation by collagenase digestion (Sigma, USA). Then, the cells were seeded in a 3.5 cm dish with 0.3×10^5^ cells using the DMEM/F12 containing 10% Fetal Bovine Serum (ThermoFisher, USA), 1% Biotin, and 1% penicillin in 5% CO_2_, and two days post confluence, we exposed the cells to DMEM/F12, 10% Fetal Bovine Serum (ThermoFisher, USA), 1% Biotin, and 1% penicillin and streptomycin containing 0.5 mM 3-isobutyl-1-methyl-xanthine (Sigma, USA), 1 µM dexamethasone (Sigma, USA), and 5 µg/mL insulin (Solarbio, China) and 1 µM rosiglitazone. After two days, the cells were maintained in a medium with 5 µg/mL insulin (Solarbio, China) and 1 µM rosiglitazone. After induction into mature adipocytes, oil red staining or cell harvesting was performed for western blot and RT-qPCR.

The aging model was seeded in a 3.5 cm dish containing 0.3×10^5^ cells and 5 µM etoposide reagent, and it was treated 48 h after collection.

### Flow cytometry

The SVF was obtained from digesting murine iWAT pads with 100 µL of a FACS buffer containing 3% bovine serum albumin in a phosphate-buffered saline (PBS). It was preincubated for 15 min with 0.5 µL of anti-CD16/32 and incubated for 15 min with 1 µL fluorochrome-labeled antibodies. DAPI was used for viability dye in this research. Flow cytometry test was done for the SVF population (CD31^-^CD45^-^PDGFRA^+^SCA1^+^), and specific antibodies are shown in Table 2.

### Plasmid construction and transfection

*Hnrnpc/Lcp1* cDNA was cloned into pCDNA 3.1(-) plasmid using standard molecular cloning methods [27]. *Lcp1-wt* and *Lcp1-mut* cDNA were cloned into psiCHECK2. When the cell density reached approximately 70%, we transfected the plasmids following the Lipofectamine 3000 (ThermoFisher, USA) protocol instructions. Following the transfection of HNRNPC and LCP1 overexpression plasmids, we initiated the SVF-induced differentiation culture process. Subsequently, fluorescence activity was detected in the *Lcp1-wt* and *Lcp1-mut* groups 48 hours after transfection, using the dual-luciferase reporter kit (Promega, USA) procedure.

### Western blot analysis

Total protein of SVF or adipocyte was extracted with cell lysis buffer (1.0 M Tris-HCl (pH = 6.8) 10 mL, 10% SDS 40 mL, volume to 200 mL with ddH_2_O). After measuring the protein concentrations by NanoPhotometer, 5×loading buffer for protein, 30 µg protein was loaded and separated on 10% SDS-PAGE gel. It was then transferred on 0.45 µm polyvinylidene fluoride (PVDF) membranes (Millipore, USA), blocked with 5% milk in TBST for 1 h at room temperature, and incubated with primary antibody overnight at 4 °C. Specific antibodies are listed in Table 2. It was then washed with TBST for 10 min for a total of three times. Then, it was incubated with the secondary antibodies (Rabbit 1:10000 Jackson, USA) at room temperature for 1 h, washed with TBST for 10 min and three times, and exposed to a chemiluminescence kit (Share Bio, China).

**Table 2:** Antibodies.

Antibody	Vendor or Source	Catalog	Working concentration
**Name**			
**a-Tubulin**	Cell Signaling Technology	#2125S	1:1000 for WB
**AKT**	Cell Signaling Technology	#9272S	1:1000 for WB
**C/EBPa**	Santa	SC-61	1:1000 for WB
**HNRNPC**	ThermoFisher	PA5-22280	1:1000 for WB
**HNRNPC**	ThermoFisher	MA1-24631	1:400 for IHC
**HSP90**	Cell Signaling Techonology	#4877S	1:1000 for WB
**p-AKT(Ser473)**	Cell Signaling Technology	#4058s	1:1000 for WB
**PPARg**	Cell Signaling Technology	#2442s	1:1000 for WB
**LCP1**	Abclonal	A5561	1:1000 for WB
**Peroxidase-AffiniPure Goat Anti-Rabbit IgG (H+G)**	Jackson	111-035-003	1:10000 for WB
**Peroxidase-AffiniPure Goat Anti-Mouse IgG (H+G)**	Jackson	115-035-003	1:10000 for WB
**Anti-N6-methyladenosine (m6A)**	Millipore	MABE1006	1:1000 for dot blot
**FITC anti-mouseCD31**	Biolegend	102406	1ug for million cells
**APC/Cyanine anti-mouse CD45**	Biolegend	103115	1ug for million cells
**PE anti-mouse CD140a**	Biolegend	135905	1ug for million cells
**Ly-6A/E (Sca-1) Mnonoclonal, PerCP-Cyanine5.5**	eBioscience	45598182	0.25ug for per test

### Reverse transcription (RT) and quantitative real-time PCR (qPCR) analysis

The total RNA was isolated with the TRIzol^TM^ reagent (Invitrogen, USA). The RevertAid First Strand cDNA Synthesis kit (ThermoFisher, USA) was used to synthesize complementary DNA (cDNA), and qPCR was performed by PowerUp^TM^ SYBR^TM^Green Master Mix (ThermoFisher, USA) in a Quantstudio3/5 (Thermo Fisher, USA) real-time PCR instrument. Expression levels of the target genes were calculated using the 2^-ΔΔCT^ method and normalized to the standard housekeeping gene 18S for relative mRNA level representation compared to the internal control. Specific primers are shown in Table 3.

### Measurement of m^6^A modification

The RNA was purified using the RNA purification kit QIAGEN (Millipore, USA). Then, it was added onto a nylon membrane for continuous cross-linking by UV light at 254 nm for 15 min, washed 2% with PBST for 5 min, and closed with 5% skimmed milk powder for 1 h. The m^6^A antibody (1:1000 Millipore, USA) underwent incubation at 4 °C overnight and was washed with 2% PBST for 5 min and three times. Then, it was incubated with the secondary antibodies (Mouse 1:10000 Jackson, USA) at room temperature for 1 h, washed thrice with 2% PBST for 5 min, and exposed to methylene blue staining. Photos were taken for documentation.

**Table 3:** Primers.

Primer Name		Primer sequences (5’-3’)
***18S***	Forward	CGCCGCTAGAGGTGAAATTCT
	Reverse	CATTCTTGGCAAATGCTTTCG
***Acc***	Forward	CACCAGTTTTGCATTGAGAAC
	Reverse	TACGCTGTTGAGTTCATAGGC
***Cdkn1α***	Forward	CCTGGTGATGTCCGACCTG
	Reverse	CCATGAGCGCATCGCAATC
***Cdkn2α***	Forward	GCTTCTCACCTCGCTTGTC
	Reverse	CGCTGCTGTACTCCCTCA
***C/ebpa***	Forward	CAAGAACAGCAACGAGTACCG
	Reverse	GTCACTGGTCAACTCCAGCAC
***Fabp4***	Forward	GCGTAAATGGGGATTTGGTC
	Reverse	CTCCTGTCGTCTGCGGTGATT
***Fasn***	Forward	AGGTGGTGATAGCCGGTATGT
	Reverse	TGGGTAATCCATAGAGCCCAG
***Gapdh***	Forward	AGGTCGGTGTGAACGGATTTG
	Reverse	TGTAGACCATGTAGTTGAGGTCA
***Hnrnpc***	Forward	ACTTGGACTATGACTTTCAACGG
	Reverse	GCTGACGTTTGGAAGGCAC
***Il-1β***	Forward	GAAATGCCACCTTTTGACAGTG
	Reverse	TGGATGCTCTCATCAGGACAG
***Il-6***	Forward	GACAACCACGGCCTTCCCTAC
	Reverse	TCATTTCCACGATTTCCCAGA
***Lcp1***	Forward	TGATGGAGCTCAGAGAGGCT
	Reverse	CTATGCCATCGCCTACAGCA
***Pparg***	Forward	CCAAATACGTTTATCTGGTGTTTC
	Reverse	CGTTGCTACATTGTCTCGC
***Srebp1c***	Forward	GGAGCCATGGATTGCACATT
	Reverse	CAGGAAGGCTTCCAGAGAGG
***Tnf-α***	Forward	CCCTCACACTCACAAACCAC
	Reverse	ACAAGGTACAACCCATCGGC
***Tpm2***	Forward	GTGGCTGAGAGTAAATGTGGG
	Reverse	TTGGTGGAATACTTGTCCGCT
***Tp53***	Forward	CCCCTGTCATCTTTTGTCCCT
	Reverse	AGCTGGCAGAATAGCTTATTGAG

### m^6^A methylated RNA immunoprecipitation

The total RNA of 3T3-L1 cells was extracted using the TRIzol^TM^ reagent, followed by the Magna MeRIP m^6^A kit (Sigma, USA). Next, 2 μL of fragmentation buffer was added to shear the RNA to 100 nt, and 30 μg of fragmental RNA was used for input with 50 μl of beads for each reaction. After washing the beads with 1×IP buffer, 10 μg anti-m^6^A antibody or 10 μg normal mouse IgG was used for the total fragmented RNA, and rotation at room temperature was done for 30 min. Then, 300 μg of fragmented total RNA with beads-antibody complex were added to the MeRIP reaction mixture, and rotation was done at 4 °C overnight. The elution buffer was used to obtain RNA for RT-qPCR, which was pulled down by the m^6^A antibody.

### RNA pull-down

The synthesized and labeled biotin containing m^6^A motif RNA sequences were incubated with lysate at 4 °C for 3 h. *Lcp1*wt-1 5’biotin-AAGCCAUUGCAAAGUCCUG AACCUUGGAGA-3’; *Lcp1*mut-1 5’biotin-AAGCC AUUGCAAAGUCCUGAGCCUUGA GA-3’; *Lcp1*wt-2 5’biotin-UUUGCUCCAUUCCCCUCAGAACCAUAC CUG-3’; *Lcp1*mut-2 5’biotin-UUUGCUCCAUUCC CCUCAGAGCCAUACCUG-3’; *Lcp1*wt -3 5’biotin-AAAGUGUAAUAAAGAGUAGAACCACUAGGG-3’; *Lcp1*mut-3 5’biotin-AAAGUGUAAUAAAGAGUAG AGCCACUAGGG-3’; *Lcp1*wt-4 5’biotin-GAUAAGC AAUUUCCACGAGAACCUACCAAA-3’; *Lcp1*mut-4 5’biotin-GAUAAGCAAUUUCCACGAGAGCCUACC AAA-3’; *Lcp1*wt-5 5’biotin-AAAGCCA CAGAGCUUC GUGAACCAAAGCAA-3’; *Lcp1*mut-5 5’biotin-AAAG CCACAGAGCUUCGUGAGCCAAAG C AA-3’. Then, 20 μL of protein A/G beads were added for each sample, washed three times, and co-incubated with RNA-lysate complexes at 4 °C overnight. The mixture was washed six times with washing buffer and added to 1×SDS-PAGE buffer at 95 °C for 5 min for western blot.

### Dual-luciferase reporter assay

Next, 3’UTR of the *Lcp1* m^6^A sequence was inserted into psiCHECK2 plasmid, and the designed plasmid was transfected into SVF. After 48 h, the cells were collected following the procedure of the dual-luciferase reporter assay system (Promega, USA). Sequences: Lcp1-wt: AAGCCAUUGCAAAGUCCUGAACCUUGGAGA; Lcp1-mut: AAGCCAUUGCAAAGUCCUGAGCCUUG GAGA.

### RNA stability assay

Actinomyces D was added to the SVF, knocking down HNRNPC, and the cells were harvested at 0, 2, and 4 h. The RNA was extracted for RT-qPCR experiments.

### Cell transfection with siRNAHnrnpc

When the SVF cell density was around 60%, the cells were transfected with siRNA*Hnrnpc* (sense 5’-3’: GC UUAGAAAUCUUAUCCCATT, antisense 5’-3’: UGGG AUAAGAUUUCUAAGCTT) according to the Lipo-fectamine RNAmax operation manual, and the cells were collected after 48 h.

### RNA-Seq

RNA was isolated and purified from total samples using TRIzol^TM^ reagent (Thermo Fisher, USA). The mRNA containing PolyA (polyadenylate) was specifically captured by two rounds of purification using oligo(dT) magnetic beads (Thermo Fisher, USA). The captured mRNA was fragmented, and the fragmented RNAs were used to synthesize cDNAs using reverse transcriptase (Invitrogen, USA). The cDNAs were then converted into DNA duplexes by two-strand synthesis using E. coli DNA polymerase I (NEB, USA) with RNase H (NEB, USA). The duplexes were doped with dUTP solution (Thermo Fisher, USA) to complement the ends of the double-stranded DNA and create flat ends. An A base was added to each end allowing the ligation to a junction with a T base at the end, and the fragment size was screened and purified using magnetic beads. The second strand was digested with UDG enzyme (NEB, USA), and then PCR was performed to generate a library with a fragment size of 300bp ± 50bp (strand-specific library). Finally, we used Illumina NovaseqTM 6000 (LC Biotechnology CO. Ltd. Hangzhou, China) to sequence it bipartite according to the standard operation, and the sequencing mode was PE150.

### RNA immunoprecipitation and RIP-Seq

HNRNPC RNA immunoprecipitation assay was performed with Magna RIP^TM^ RNA-Binding Protein Immunoprecipitation Kit (Millipore, USA). Briefly, 50 μL of protein A/G beads (Millipore, USA) were used for each sample and washed three times. Then, 5 μg of anti-HNRNPC antibody (ThermoFisher, USA) was placed in the lysis and washing buffer with the beads for 30 min at room temperature. The lysate containing the bead-antibody complex was then incubated in the RIP immunoprecipitation buffer at 4 °C overnight, and 10% of the input lysate was removed. Normal rabbit IgG was used as a negative control (Millipore, USA). The complexes were washed six times and added to TRIzol^TM^ reagent to extract RNA. The integrity of the sample total RNA was tested using agarose gel electrophoresis, and it was quantified and further quality-controlled using a NanoDrop ND-1000. For sequencing library construction, 1-2 μg of total RNA per sample was selected. The total RNA was first subjected to mRNA enrichment using the NEB Next Poly(A) mRNA Magnetic Isolation Module. The processed RNA product was then used to construct the library using the KAPA Stranded RNA-Seq Library Prep Kit (Illumina). After construction, the libraries were quality-controlled using an Agilent 2100, quantified by qPCR, and clustered libraries were sequencing runs on an Illumina NovaSeq 6000 by AKsomics (Shanghai, China).

### Data analyses

For RNA-seq data analysis, raw data (raw reads) of FASTQ format were filtered by Cutadapt (v1.9) to get high-quality clean reads. We aligned reads of all samples to the mouse reference genome GRCm38 using HISAT2(v2.2.1). The mapped reads of each sample were assembled using StringTie (v2.1.6) with default parameters. After the final transcriptome was generated, StringTie and Ballgown were used to estimate the expression levels of all transcripts and perform expression abundance for mRNAs by calculating the FPKM (fragment per kilobase of transcript per million mapped reads) value. Genes differential expression analysis was performed by DESeq2 software between two different groups. The genes with the false discovery rate (FDR) parameter below 0.05 and |log_2_ fold-change| > 1 were considered differentially expressed genes.

For RIP-seq data analysis, all input and IP samples’ raw sequencing data were preprocessed using Cutadapt (v1.9). The filtered reads of all samples were aligned to the mouse reference genome GRCm38 using HISAT2. (v2.2.1). For calculating the relative HNRNPC binding level for each gene, StringTie (v2.1.6) was used to assemble the transcripts and estimate the expression levels of all transcripts, and the relative expression levels of the transcript represent the HNRNPC binding level of the transcript. Subsequent differential expression analysis was performed using Ballgown based on transcripts with an average FPKM greater than 0.5 in the sample. We identified high-confidence HNRNPC binding genes by setting up the cutoff line based on log_2_ fold-change for IP versus input > 5 and p-value < 0.05. The ggplot2 package creates a Venn diagram, volcano, bubble, and petal plot.

### Data availability

The raw sequence datas have been submitted to the NCBI Short Read Archive (SRA) with accession number with accession number PRJNA1101485 and PRJNA1101890.

### Ethics approval and consent to participate

Human tissue sampling procedures and operations were approved by the Ethic Committee of Chengdu No. 3 Hospital (Permit Number: No. 20231S-177). Animal experimental procedures and operations were approved by the Animal Welfare and Ethics Committee of Chongqing Medical University (Permit Number: IACUC-CQMU-2023-0156).

### Statistics

All data points are quantitative and overlaid with mean ± SD. The Shapiro-Wilk normality test was employed to assess the normality of the data. The unpaired/ paired two -tailed Student’s *t*-test was used to analyze the comparisons between the two groups. A non-parametric test, specifically the Mann-Whitney test, is employed when the data does not adhere to a normal distribution. A one-way analysis of variance (ANOVA) was used to determine statistically significant differences between groups. Data from *in vitro* experiments were analyzed in at least three replicate experiments. The gray values of the western blot were quantified with ImageJ software. GraphPad Prism 9 software was also used. P values are two significant figures. P<0.05 was considered statistically significant.

**Figure 1.**
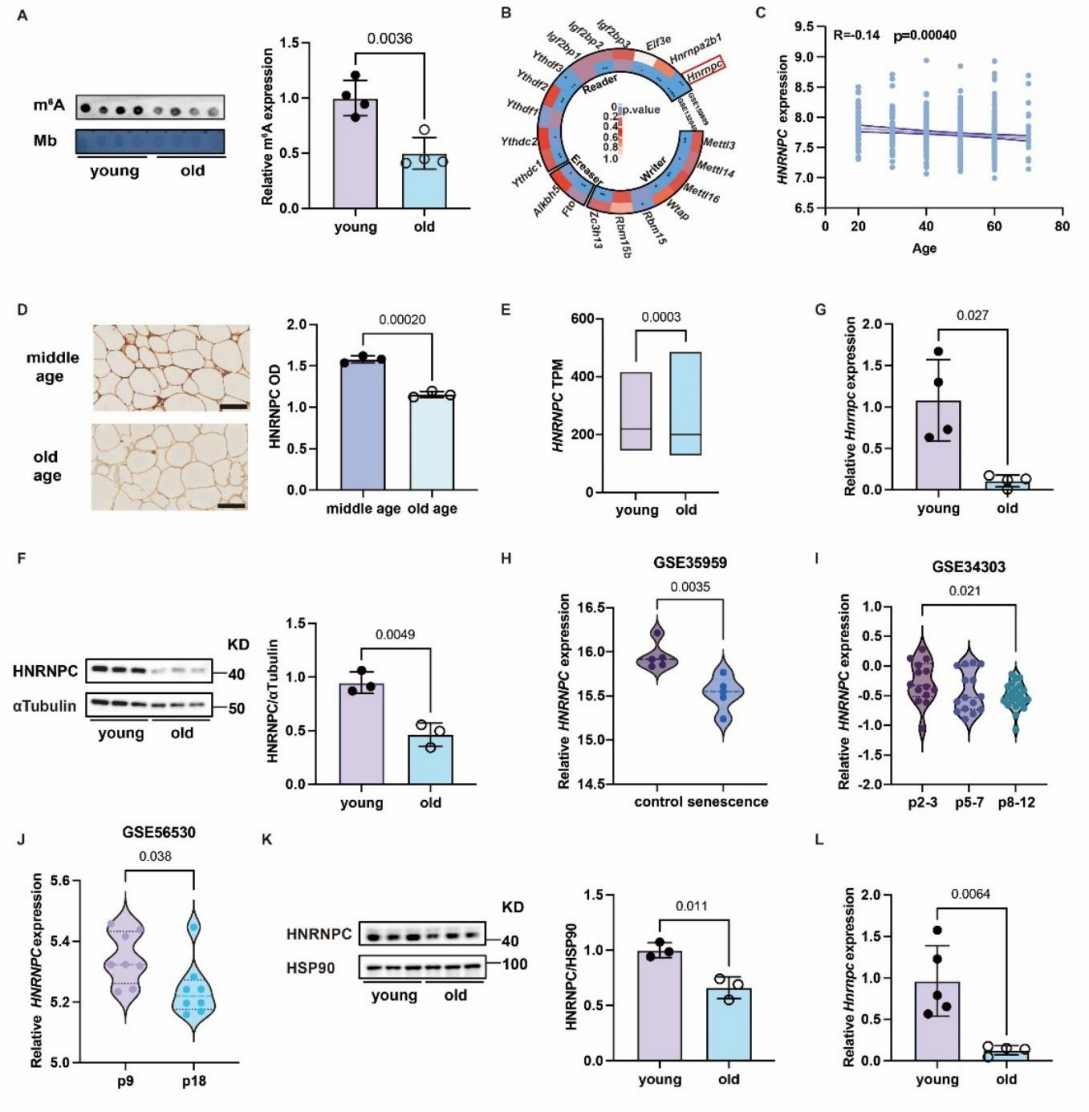
**The level of m^6^A modification and HNRNPC in adipose tissue during aging**. (**A**) The expression of m^6^A modification in young (n = 4) and aging adipose tissue (n = 4). (**B**) The expression of m^6^A regulators in the GEO databases (GSE132040 and GSE159809). (**C**) Correlation of the expression of *HNRNPC* with age (www.proteinatlas.org/). (**D**) Immunohistochemistry detection of HNRNPC in human middle-aged (n = 3) and old-aged adipose tissue (n = 3). Scale bars, 100 µm. (**E**) The expression of HNRNPC in young (n=110) and old (n=237) human adipose tissue (www.proteinatlas.org/). (**F**) Western blot analysis of HNRNPC in young (n = 3) and aging adipose tissue (n = 3) in mice. G. RT-qPCR analysis of *Hnrnpc* in young (n = 4) and aging adipose tissue (n = 4). (**H**) The expression of *Hnrnpc* in control (n = 5) and senescence (n = 5) of human mesenchymal stem cells (GSE35959). (**I**) The expression of *HNRNPC* in p2-3 (n = 14), p5-7 (n = 15) and p8-12 (n = 22) of human mesenchymal stem cells (GSE34303). (**J**) The expression of *Hnrnpc* in p9 (n = 8) and p18 (n = 8) of human mesenchymal stem cells (GSE56530). (**K**) Western blot analysis of HNRNPC in young (n = 3) and aging SVF (n = 3). (**L**) RT-qPCR analysis of *Hnrnpc* in young (n = 5) and aging SVF (n = 4). Mb, methylation blue, p2-3, passage 2-3, p5-7, passage 5-7, p8-12, passage 8-12, p9, passage 9, p18, passage 18. All data were shown as mean ± SD. After performing the Shapiro-Wilk normality test to examine the normal distribution, the unpaired, two-tailed Student's t-test with Welch’s correction or non-parametric Mann-Whitney test was utilized to assess the significance between the two groups. P < 0.05 was considered statistically significant.

**Figure 2.**
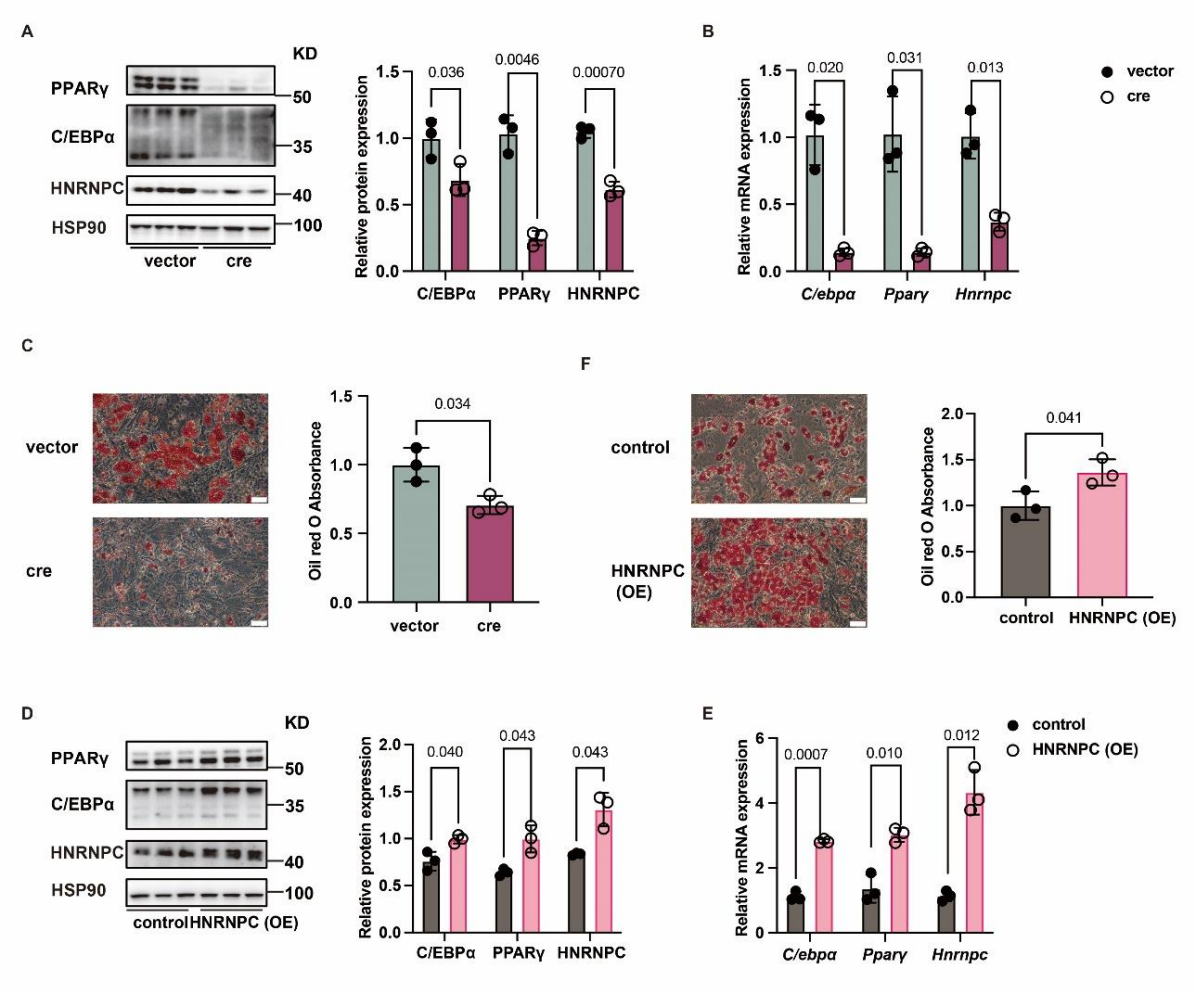
**HNRNPC promotes adipogenesis *in vitro***. (**A**) Western blot analysis of C/EBPα, PPARγ and HNRNPC in vector (n = 3) and cre virus-treated SVF (n = 3). (**B**) RT-qPCR analysis of *C/ebpα*, *Pparγ* and *Hnrnpc* in vector (n = 3) and cre virus-treated SVF (n = 3). (**C**) The oil red staining in vector (n = 3) and cre virus-treated SVF (n = 3). Scale bars, 50 μm. (**D**) Western blot analysis of C/EBPα, PPARγ and HNRNPC in control (n = 3) and HNRNPC-transfected SVF (n = 3). (**E**) RT-qPCR analysis of *C/ebpα*, *Pparγ*, and *Hnrnpc* in control (n = 3) and HNRNPC-transfected SVF (n = 3). (**F**) The oil red staining in control (n = 3) and HNRNPC-transfected SVF (n = 3). Scale bars, 50 μm. All data were shown as mean ± SD. After performing the Shapiro-Wilk normality test to examine the normal distribution, the unpaired, two-tailed Student's t-test with Welch’s correction was utilized to assess the significance between the two groups. P < 0.05 was considered statistically significant.

## RESULTS

### The expression of HNRNPC is positively associated with reduced adipogenesis during adipose tissue aging

To investigate whether m^6^A modification changes in adipose tissue with age, we used a m^6^A dot blot to detect m^6^A levels of total RNA in the adipose tissues of young and old mice. Compared to old mice, young mice had higher levels of m^6^A in their adipose tissue (Fig. 1A). By studying the GEO databases (GSE132040 and GSE159809), the expression of m^6^A modification-associated genes was higher in young mice compared to old mice. Also, the expression differential of HNRNPC was the most significant gene in humans and mice (Fig. 1B). Moreover, the expression level of HNRNPC showed a significant negative correlation with age (Fig. 1C). We then used immunohistochemistry to detect the HNRNPC expression in the subcutaneous adipose tissues of middle-aged and elderly people. The results showed that HNRNPC was lowly expressed in the aged group (Fig. 1D). The mRNA level of *HNRNPC* was similarly significantly reduced in the aging human (Fig. 1E). The protein and mRNA levels of HNRNPC in subcutaneous adipose tissue of young mice were higher than in aged mice (Fig. 1F-G). Adipose tissue is mainly composed of adipocytes and stromal vascular fraction (SVF), and each has different functions. To further investigate the function of HNRNPC in adipose tissue during aging, we examined HNRNPC expression in primary adipocyte and SVF. The *HNRNPC* mRNA expression in human SVF showed a significant increase compared with that of adipocytes (GSE135776). The protein expression of HNRNPC in SVF showed an increasing trend and significantly increased mRNA level compared to adipocytes in mice. (Supplementary Fig. 1A-C). Considering that the main role of SVF is to generate new adipocytes, we hypothesized that HNRNPC may be involved in the adipogenesis of adipose tissue during aging. To further identify the relationship between HNRNPC expression and adipogenesis, we observed a significant decrease in HNRNPC mRNA levels in senescent mesenchymal stem cells derived from human adipose tissue (Fig. 1H-J). Additionally, through western blot and RT-qPCR experiments, we demonstrated that the expression of HNRNPC in SVF was higher in young mice than in old mice (Fig. 1K-L). Moreover, the same results were obtained in aged SVF that was treated with etoposide [28] (Supplementary Fig. 1D-E). In addition, as 3T3-L1 cells differentiate into mature adipocytes, the expression pattern of HNRNPC coincides with a specific phase of the C/EBP family. This observation implies a potential regulatory role for HNRNPC in adipocyte differentiation (Supplementary Fig. 1F). Our data suggest that the low expression of HNRNPC may be associated with reduced adipogenesis during aging.

**Figure 3.**
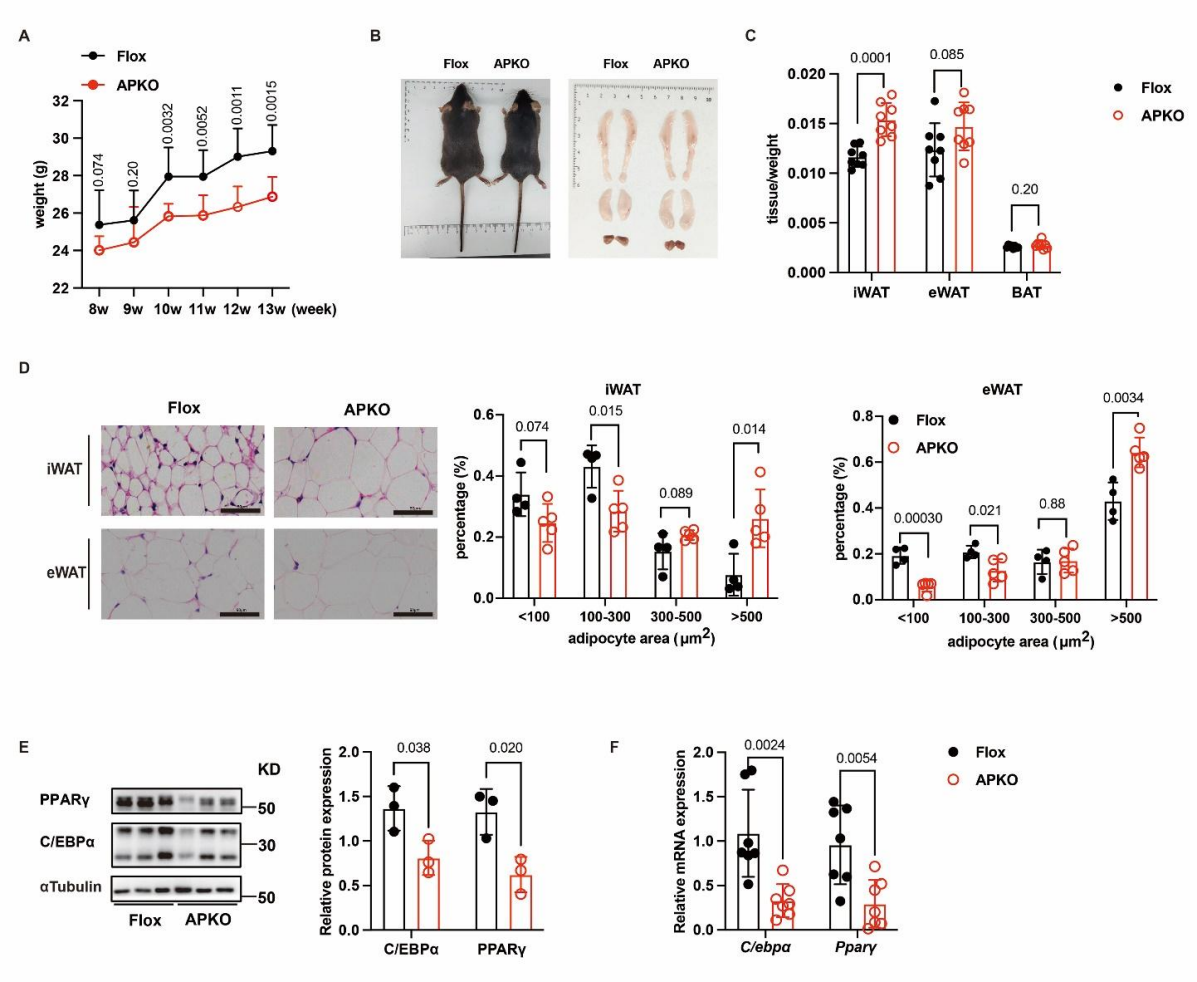
**Knocking down HNRNPC inhibits adipogenesis in mice**. (**A**) The weekly weight of Flox (n = 8) and APKO (n = 8) mice under normal feed. (**B**) The photograph of general and tissue of Flox and APKO mice. (**C**) The tissue/weight of Flox (n = 8) and APKO (n = 8) mice. (**D**) The H&E staining of iwat and ewat of Flox (n = 4) and APKO (n = 5) mice. Scale bars, 50 μm. (**E**) The protein expression of C/EBPα and PPARγ in Flox (n = 3) and APKO (n = 3) mice. (**F**) The mRNA expression of *C/ebpα* and *Pparγ* in Flox (n = 7) and APKO (n = 7). All data were shown as mean ± SD. After performing the Shapiro-Wilk normality test to examine the normal distribution, an unpaired two-tailed Student′s t-test or unpaired two-tailed Student′s t-test with Welch’s correction was utilized to assess the significance between the two groups. P < 0.05 was considered statistically significant.

**Figure 4.**
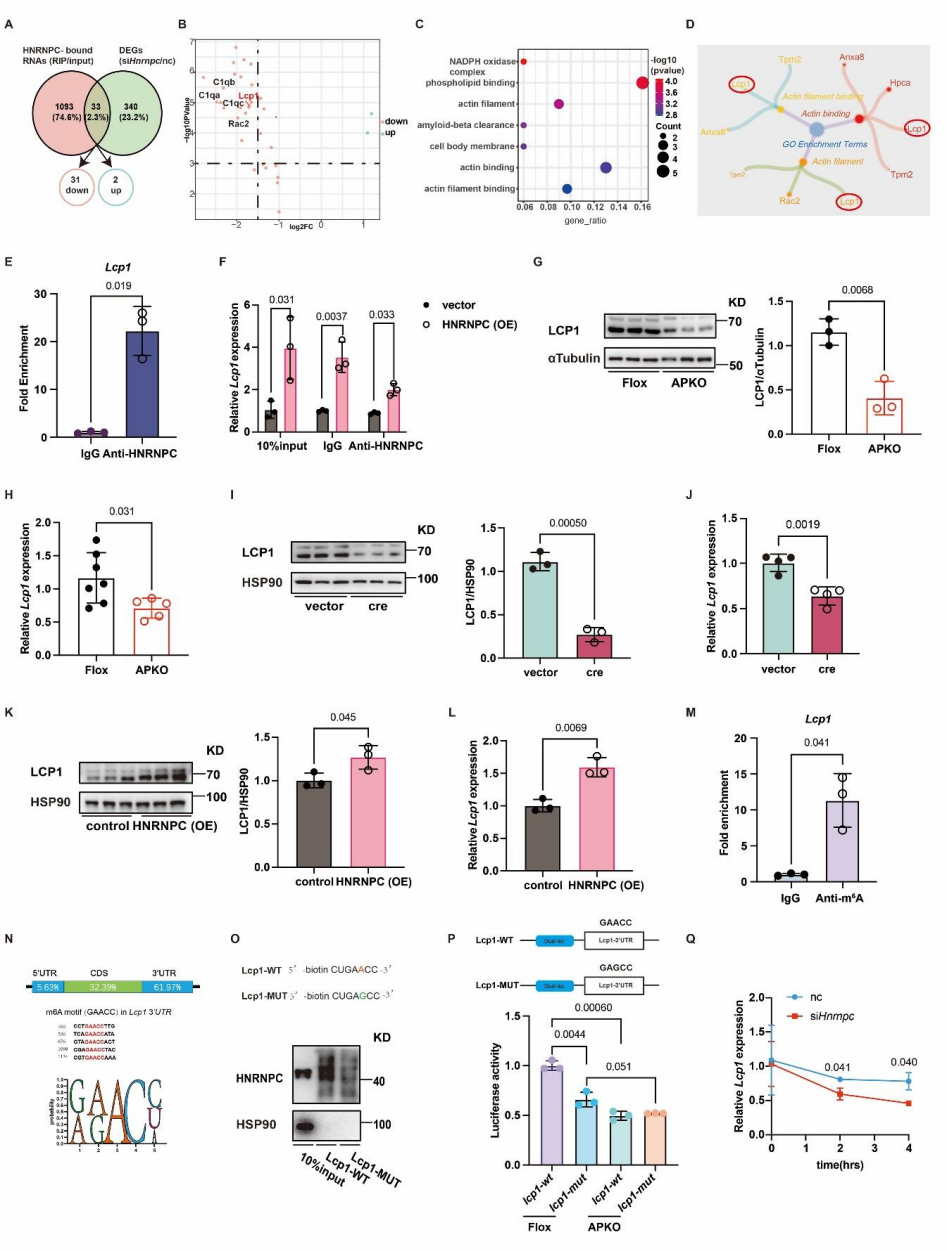
**HNRNPC regulates *Lcp1* mRNA stability through binding to the m^6^A motif of *Lcp1***. (**A**) The Venn plot of RNA-Seq (knocking down HNRNPC by transfecting siRNA *Hnrnpc* in svf) and RIP-Seq (using anti-HNRNPC to enrich RNA). (**B**) The Volcano plot of overlap gene. (**C**) The GO class of overlap gene. (**D**) The petal plot of the actin-related term. (**E**) The expression of *Lcp1* in RIP-qPCR with anti-IgG (n = 3) and anti-HNRNPC (n = 3). (**F**) The expression of *Lcp1* in RIP-qPCR (vector (n = 3) and overexpression of HNRNPC (n = 3)) with anti-HNRNPC. (**G**) The protein expression of LCP1 in Flox (n = 3) and APKO (n = 3) mice. (**H**) The mRNA expression of *Lcp1* in Flox (n = 7) and APKO (n = 5) mice. (**I**) The protein expression of LCP1 in vector (n = 3) and cre virus-treated SVF (n = 3). (**J**) The mRNA expression of *Lcp1* in vector (n = 4) and cre virus-treated SVF (n = 4). (**K**) Western blot analysis of LCP1 in vector (n = 3) and HNRNPC-transfected SVF (n = 3). (**L**) RT-qPCR analysis of *Lcp1* in vector (n = 3) and HNRNPC-transfected SVF (n = 3). (**M**) The expression of *Lcp1* in MeRIP-qPCR with anti-IgG (n = 3) and anti-m^6^A (n = 3). (**N**) The percentage of m^6^A motif in *Lcp1* and m^6^A motif (GAACC) in (https://rna.sysu.edu.cn/rmbase/). (**O**) The western blot of HNRNPC in RNA-Pull down. (**P**) The dual luciferase reporter assay of *Lcp1*-wt or *Lcp1*-mut in Flox (n = 3) and APKO (n = 3) group. (**Q**) The expression of *Lcp1* in HNRNPC deficiency with actinomycin D, nc (n = 3), si*Hnrnpc* (n = 3). All data were shown as mean ± SD. After performing the Shapiro-Wilk normality test to examine the normal distribution, the unpaired, two-tailed Student's t-test with Welch’s correction was utilized to assess the significance between the two groups. One-way analysis of variance (ANOVA) was employed to analyze the data pertaining to multiple groups. Subsequently, multiple comparisons were conducted using the uncorrected Fisher's LSD test. P < 0.05 was considered statistically significant.

### HNRNPC deficiency represses adipogenesis in vitro

To explore the role of HNRNPC in adipogenesis, we used the cre adenovirus to knock down HNRNPC in SVF obtained from the subcutaneous adipose tissue of *Hnrnpc^flox/flox^* mice, which were treated with adipogenesis-inducing supplements. C/EBPα and PPARγ, the critical transcriptional regulation factors of adipogenesis, showed decreased protein and mRNA levels (Fig. 2A-B). Moreover, the oil red staining results showed reduced staining intensity in SVF with HNRNPC knockdown (Fig. 2C). In contrast, HNRNPC overexpression promoted the protein and mRNA expression levels of C/EBPα and PPARγ (Fig. 2D-E). Meanwhile, the oil red staining results revealed increased staining intensity in HNRNPC-overexpressed SVF (Fig. 2F). These findings suggest that HNRNPC mediates adipogenesis and promotes adipogenesis *in vitro*.

### HNRNPC deficiency inhibits adipogenesis in mice

To further evaluate the *in vivo* physiological function of HNRNPC in adipogenesis, we generated an adipocyte progenitor-specific HNRNPC knockdown (*Hnrnpc-APKO*) mouse by crossing *Hnrnpc^flox/flox^* mice with the transgenic mice harboring Cre recombinase driven by the pdgfrα promoter (pdgfrα-cre). Mice littermates without the cre gene (heterozygote *Hnrnpc-Flox*) were used as the control group (Supplementary Fig. 2A). Western blot results demonstrated a dramatic reduction of HNRNPC protein levels in subcutaneous inguinal WAT (iWAT) and visceral epidermal WAT (eWAT) but not in other tissues compared to *Hnrnpc-Flox* mice, thus verifying the specific knockdown of HNRNPC in adipocyte progenitor (Supplementary Fig. 2B).

Firstly, we analyzed the body weights of *Hnrnpc-Flox* and *Hnrnpc-APKO* mice fed a chow diet for 6 weeks (age 13 weeks). The results revealed that the *Hnrnpc-APKO* mice gained less body weight than *Hnrnpc-Flox* mice under chow-fed conditions (Fig. 3A). However, the former had bigger iWAT and eWAT, and they weighed more than *Hnrnpc-Flox* mice (Fig. 3B-C). Histological analysis showed that adipocytes were bigger in *Hnrnpc-APKO* mice than in *Hnrnpc-Flox* mice, while the histological morphology of BAT has no difference (Fig. 3D and Supplementary Fig. 2C). Western blot and RT-qPCR results exhibited that protein and mRNA expression levels of C/EBPα and PPARγ in *Hnrnpc-Flox* mice were higher than in *Hnrnpc-APKO* mice (Fig. 3E-F). To explore the effects of HNRNPC on the number of adipocyte progenitor cells, we labeled CD31^-^CD45^-^Pdgfrα^+^Scα1^+^ flow antibodies. The results showed lower proportions of adipocyte progenitor in the *Hnrnpc-APKO* mice compared to *Hnrnpc-Flox* mice (Supplementary Fig. 2D). These results indicated that HNRNPC deletion inhibits adipogenesis in mice.

### LCP1 is the potential target of HNRNPC

To investigate the mechanisms underlying HNRNPC-mediated adipogenesis, we conducted a co-analysis of RNA immunoprecipitation sequencing and RNA sequencing data of SVF. This integrated approach revealed 1093 HNRNPC binding mRNAs, which were defined as high-confidence HNRNPC targets meeting the criteria of a log2 fold change (log2FC) greater than 5 enriched in IP sample versus input sample. In addition, the RNA-Seq data yielded 340 differentially expressed genes, with a log2 fold change (log2FC) greater than 1 or less than or equal to -1. Combined the RIP-Seq and RNA-Seq data, we ultimately identified 33 HNRNPC-bound transcripts, which also differencially expressed in the HNRNPC knockout group. The data demonstrated that actin-related genes were enriched in the cellular component and molecular function by further analyzing differentially expressed genes. A previous report found that the shape of the nucleus during adipogenic and osteogenic differentiation was affected by the actin cytoskeleton. During heart failure, HNRNPC physically interacts with myocardial ganglion proteins FHL2, PDLIM5, and MYH7 in the heart. Additionally, changes in the nuclear localization of HNRNPC are observed in response to changes in cellular stretch and skeleton tension [29]. These findings suggest the potential for reciprocal regulation between HNRNPC and the cytoskeleton. Therefore, we speculated the actin cytoskeleton-related gene could be targeted by HNRNPC. To investigate this, we analyzed GO enrichment terms, explicitly focusing on actin filament binding, actin binding, and actin filament. Interestingly, the result showed the involvement of LCP1 and TPM2 in these three terms (Fig. 4A-D). To ascertain whether LCP1 serves as a target gene of HNRNPC in adipogenesis, we conducted RIP-qPCR analysis. The results revealed increased *Lcp1* enrichment in the HNRNPC immunoprecipitated group and higher enrichment in the overexpression of HNRNPC group (Fig. 4E and F). Reduced LCP1 protein and mRNA levels were observed in the *Hnrnpc-APKO* mice compared with *Hnrnpc-Flox* mice. Moreover, western blot and RT-qPCR analysis showed lower LCP1 protein and mRNA expression levels in knockdown HNRNPC with cre adenovirus-treated mice than in the control group (Fig. 4G-J). Increased LCP1 protein and mRNA levels were exhibited in the overexpression of the HNRNPC group compared with the control group (Fig. 4K and L). However, the mRNA expression of *Tpm2* didn’t change with HNRNPC (Supplementary Fig. 3A-C). Taken together, our findings indicate that HNRNPC regulates LCP1 expression.

**Figure 5.**
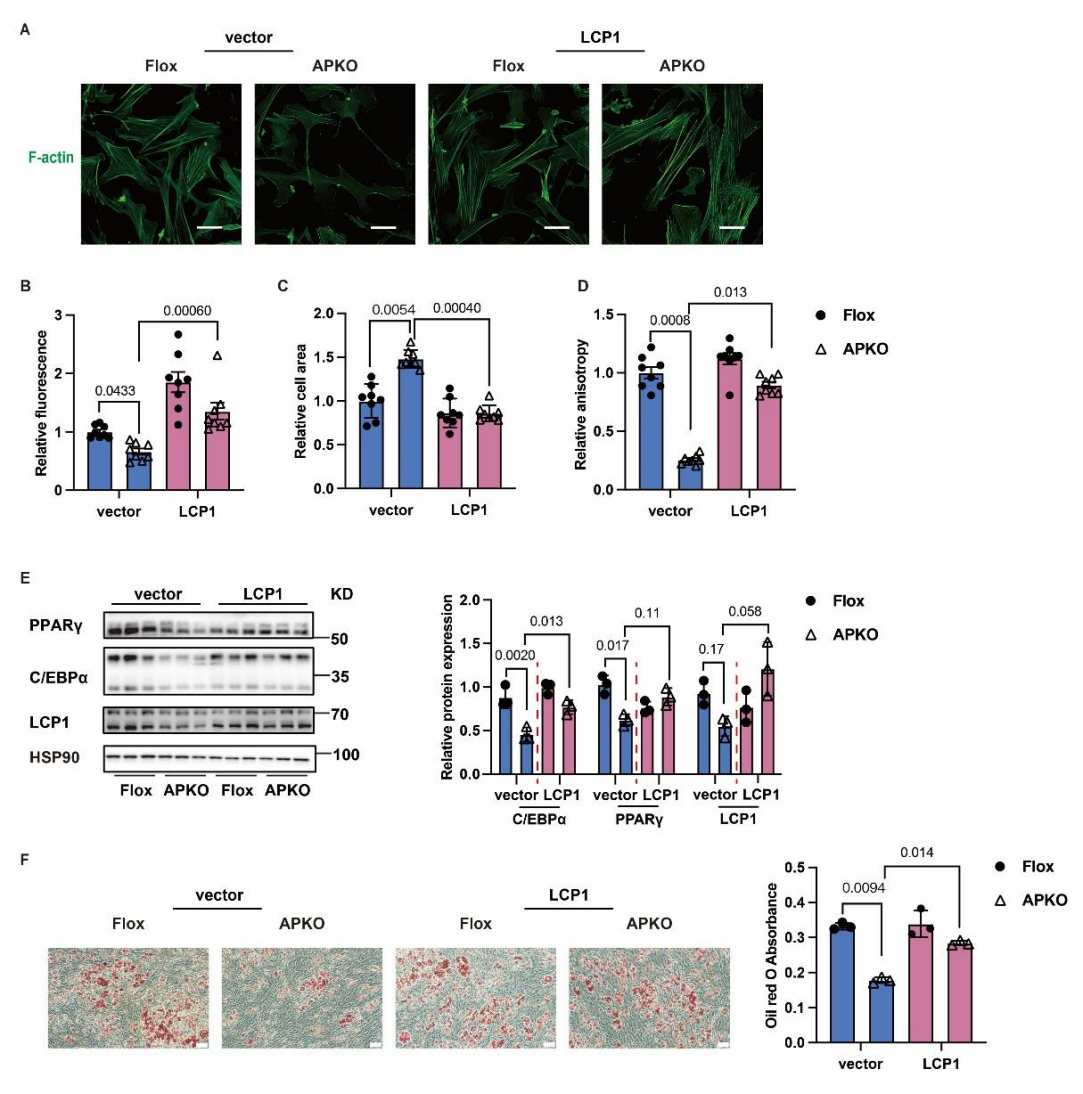
**HNRNPC modulates adipogenesis through LCP1 mediated remodeling of F-actin morphology**. (**A-D**) The F-actin fluorescence colocalization, the relative fluorescence, the relative cell area, and the relative anisotropy in four groups (Flox + vector, (n=8), APKO + vector, (n=8), Flox + LCP1, (n=8), APKO + LCP1, (n = 8)) (E) the protein expression of C/EBPα and PPARγ in four groups (Flox + vector, (n=3), APKO + vector, (n=3), Flox + LCP1, (n=3), APKO + LCP1, (n=3)). (**F**) The oil red staining in four groups (Flox + vector, (n=3), APKO + vector, (n=3), Flox + LCP1, (n=3), APKO + LCP1, (n=3)). Scale bars, 50 μm. All data were shown as mean ± SD. After performing the Shapiro-Wilk normality test to examine the normal distribution, one-way analysis of variance (ANOVA) was employed to analyze the data pertaining to multiple groups. Subsequently, multiple comparisons were conducted using the uncorrected Fisher's LSD test. *P* < 0.05 was considered statistically significant.

**Figure 6.**
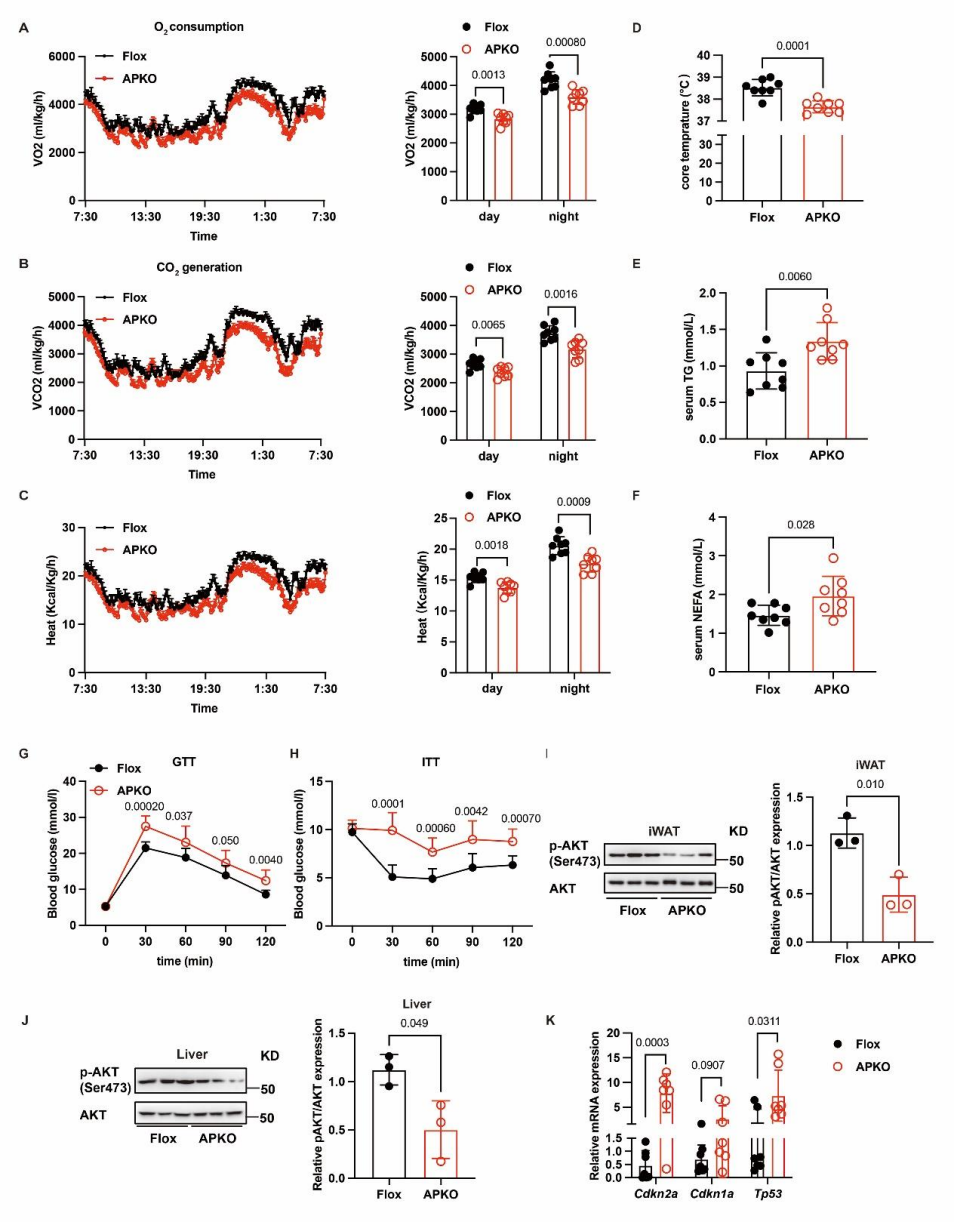
**HNRNPC deficiency decreases energy expenditure and insulin sensitivity in mice**. (**A**) The oxygen consumption of Flox (n = 8) and APKO mice (n = 8). (**B**) The carbon dioxide generation of Flox (n = 8) and APKO (n = 8) mice, n = 8:8. (**C**) The energy heat generation of Flox (n = 8) and APKO (n = 8) mice. (**D**) The core temperature of Flox (n = 8) and APKO (n = 8) mice. (**E-F**) The levels of triglycerides (TG) and non-esterified fatty acids (NEFA) of Flox (n = 8) and APKO (n = 8) mice. (**G**) The glucose tolerance test (GTT) of Flox (n = 8) and APKO (n = 8) mice. (**H**) The insulin sensitivity test (ITT) of Flox (n = 8) and APKO (n = 8) mice. (**I-J**) The p-AKT(Ser473) and AKT protein expression in iWAT and Liver of Flox (n = 3) and APKO (n = 3) mice. (**K**) RT-qPCR analysis of *Cdkn2a, Cdkn1a and Tp53* in Flox (n=7) and APKO (n=7) mice. All data were shown as mean ± SD. After performing the Shapiro-Wilk normality test to examine the normal distribution, an unpaired two-tailed Student′s t-test or unpaired two-tailed Student′s t-test with Welch’s correction was utilized to assess the significance between the two groups. P < 0.05 was considered statistically significant.

### HNRNPC stabilises Lcp1 mRNA by binding to the m^6^A motif of Lcp1

Previous studies have shown that HNRNPC plays an important m^6^A regulator role in RNA stability [30, 31]. Therefore, we wondered whether HNRNPC could regulate LCP1 expression in an m^6^A-dependent manner. Methylation RNA immunoprecipitation (Me-RIP) assay showed that the m^6^A antibody was more significantly bound to *Lcp1* than the IgG control (Fig. 4M). By using a website to predict the m^6^A-binding sites of *Lcp1*, we found that the m^6^A motifs were enriched in 3’UTR of *Lcp1*. To further clarify the binding of HNRNPC to the m^6^A motif of *Lcp1* RNA, we statistically analyzed the sequence characteristics of m^6^A on *Lcp1* 3’UTR. GAACC was the dominant consensus sequence (Fig. 4N). Then, the sequence of *Lcp1* m^6^A motif (GAACC) and *Lcp1* m^6^A mutation motif (GAGCC) with 5’biotin was designed by combining a motif score and the sample source. RNA pull-down results showed decreased HNRNPC protein expression in *Lcp1* m^6^A mutation, thus demonstrating that HNRNPC binds to the m^6^A-modified *Lcp1* (Fig. 4O and S3D). The dual-luciferase reporter assay further identified that the luciferase activity of the *Hnrnpc-APKO* group was lower than that of the *Hnrnpc-Flox* group. Meanwhile, the luciferase activity of the pSicheck2-*Lcp1*-mut group was lower than the pSicheck2-*Lcp1*-wt group (Fig. 4P). Finally, after actinomycin D treatment, HNRNPC deficiency reduced the stability of *Lcp1* mRNA (Fig. 4Q and Supplementary Fig. 3E). In conclusion, HNRNPC maintains the stability of *Lcp1* mRNA via binding to the m^6^A motif of *Lcp1*.

### HNRNPC modulates adipogenesis through LCP1 mediated remodeling of F-actin morphology

According to the sequencing results, actin filament-related processes were the major pathway affected by HNRNPC reduction in SVF. Previous research has shown that actin regulates cell shape and spreading, affecting cell differentiation [32]. This prompted us to investigate the necessity of F-actin in the adipogenesis processes during HNRNPC depletion. The phalloidin staining experiment was conducted to explore the character of F-actin in HNRNPC depletion. The relative fluorescence was decreased in HNRNPC depletion samples compared with the control group, while the relative cell area was increased in HNRNPC depletion samples. In addition, F-actin stress fibers showed decreased anisotropy and were broken in HNRNPC depletion conditions. LCP1 overexpression rescued the F-actin disruption caused by HNRNPC loss, as evidenced by cell fluorescence, cell area, and anisotropy (Fig. 5A-D). In addition to phalloidin staining results, western blot results demonstrated an increased protein expression level of C/EBPα and PPARγ when LCP1 was overexpressed under HNRNPC depletion condition (Fig. 5E). The results of the oil red staining were consistent with the above findings. (Fig. 5F). These data indicate that HNRNPC controls adipogenesis through LCP1-mediated F-actin processes.

### HNRNPC deletion leads to metabolic disorders in mice

Changes in adipose tissue can affect energy expenditure [33]. Our previous finding showed that the *Hnrnpc-APKO* mice had a bigger WAT mass than the *Hnrnpc-Flox* mice under the chow diet treatment. Therefore, we speculated that HNRNPC deficiency might influence the energy expenditure in mice. To test our hypothesis, the oxygen consumption, carbon dioxide generation, and energy heat generation of the *Hnrnpc-APKO* mice and *Hnrnpc-Flox* mice were monitored for 24h (light and dark cycles) by placing the mice in a metabolic cage at room temperature. The data revealed that the *Hnrnpc-APKO* mice showed inhibited oxygen consumption, carbon dioxide generation, and energy heat generation during light and dark cycles (Fig. 6A-C). Consistent with a reduced metabolic rate, the *Hnrnpc-APKO* mice had lower core body temperatures at room temperature compared with the *Hnrnpc-Flox* mice (Fig. 6D). In addition, the plasma levels of triglycerides (TG) and non-esterified fatty acids (NEFA) in the mice were measured. The levels of TG and NEFA were higher in *Hnrnpc-APKO* mice compared with *Hnrnpc-Flox* mice (Fig. 6E-F). Next, the glucose and insulin tolerance of chow-fed *Hnrnpc-Flox* mice and *Hnrnpc-APKO* mice were tested. After glucose injection, the blood glucose levels of *Hnrnpc-APKO* mice remained distinctly high compared to *Hnrnpc-Flox* mice. The insulin tolerance test results also showed that the *Hnrnpc-APKO* mice had attenuated insulin sensitivity (Fig. 6G-H and S4A-B). Moreover, the protein expression of AKT and pAKT (Ser473) in iWAT and Liver were tested by western blot, which further indicated that the insulin sensitivity deteriorated in *Hnrnpc-APKO* mice (Fig. 6I-J). The expression levels of aging-related genes, *Cdkn2a, Cdkn1a, and Tp53* were analyzed by RT-qPCR in both *Hnrnpc-Flox* and *Hnrnpc-APKO* mice. Notably, *Cdkn2a and Tp53* exhibited significantly elevated expression levels in *Hnrnpc-APKO* mice, while the expression of *Cdkn1a* also demonstrated an increasing trend in the absence of *Hnrnpc* (Fig. 6K). We also analyzed the expression of lipogenesis genes (*Acc*, *Fabp4*, *Srebp4*, and *Fasn*) as well as inflammation-related genes (*Tnf-a*, *Il-6*, and *Il-1β*) in the inguinal adipose tissue of *Hnrnpc-APKO* and *Hnrnpc-flox* mice. Our findings revealed that the *Hnrnpc-APKO* mice exhibited significantly reduced expression of lipogenesis-related genes, while showing a substantial increase in the expression of the inflammation regulator *Il-1β* compared to the flox mice (Supplementary Fig. 4C and D). These results suggest that HNRNPC deletion reduces overall metabolic levels, which are similar to the findings in aging mice.

### The abundances of HNRNPC and LCP1 increase following anti-aging intervention during aging

**Figure 7.**
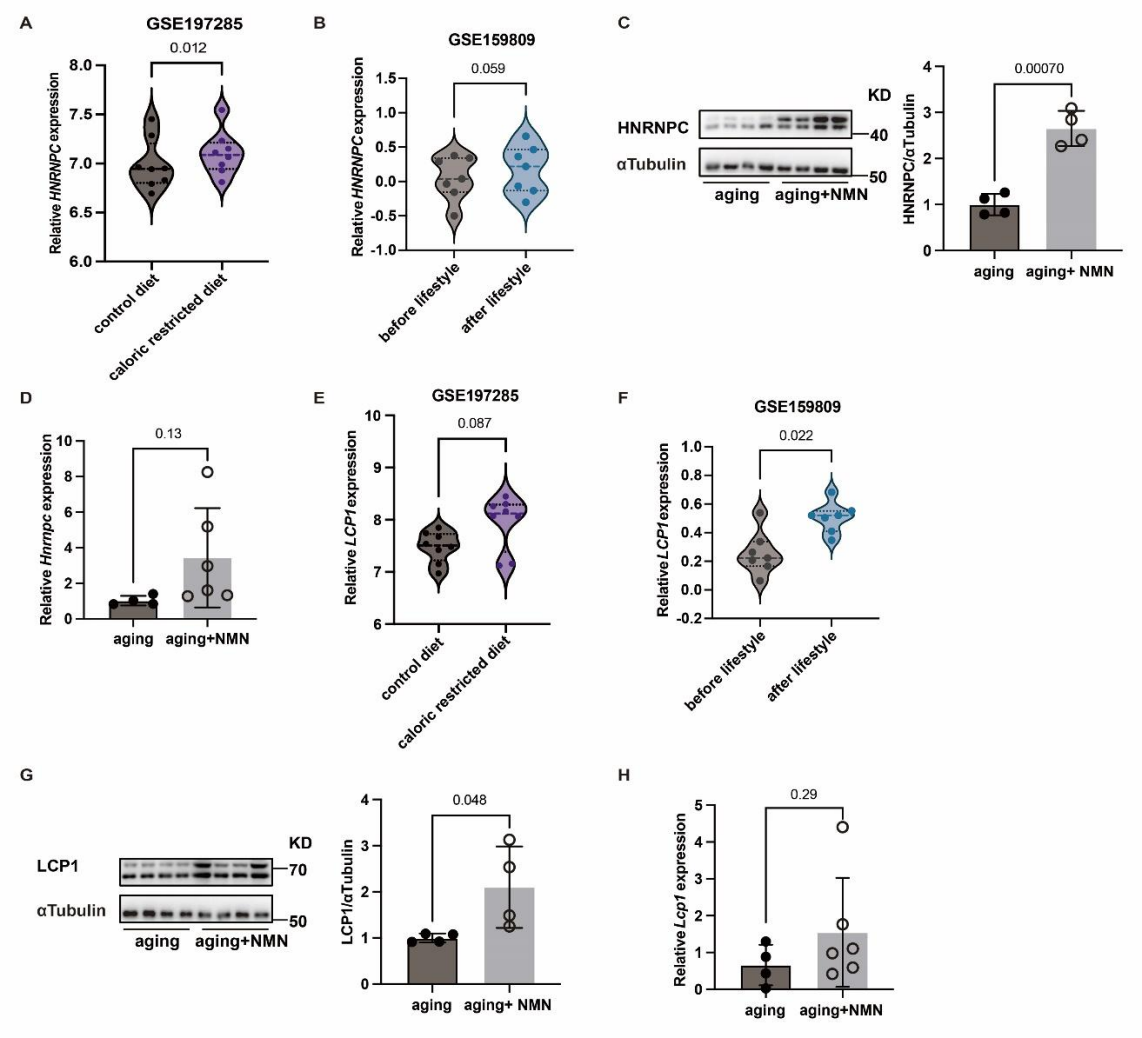
**The abundance of HNRNPC and LCP1 increases after improved metabolism in aging**. (**A-B**) The expression of *HNRNPC* in GSE197285 and GSE159809. (**C-D**) The expression of HNRNPC in adipose tissue of 24-month-old mice treated for 8 weeks with water containing 0.5 g/kg/bw.d NMN. E-F. The expression of *LCP1* in GSE197285 and GSE159809. G-H. The expression of LCP1 in adipose tissue of 24-month-old mice treated for 8 weeks with water containing 0.5 g/kg/bw/d NMN. All data were shown as mean ± SD. After performing the Shapiro-Wilk normality test to examine the normal distribution, a paired two-tailed Student′s t-test or unpaired two-tailed Student′s t-test with Welch’s correction was utilized to assess the significance between the two groups. P < 0.05 was considered statistically significant.

Adopting alterations in diet and incorporating appropriate physical activity have been recognized as effective strategies to improve metabolism and decelerate aging [34-36]. As evident in *Hnrnpc-APKO* mice, in humans, a marked elevation in the expression of *Cdkn2a* and *Tp53* genes was observed in older individuals compared to their younger counterparts. *Cdkn1a* also demonstrated an increased trend in aged subjects. At the same time, lipogenesis-related genes (*ACC*, *FABP4*, *SREBP4*, and *FASN*) were significantly reduced, and the the expression of inflammation-related genes (*TNF-a*, *IL-6*, and *IL-1β*) were increased in the subcutaneous adipose tissue of the elderly compared to the young. (Supplementary Fig. 4E-G). Notably, the mRNA level of HNRNPC in human adipose tissue showed a significant increase following a calorie-restricted diet (Fig. 7A). In addition, the expression of HNRNPC in adipose tissue was significantly up-regulated with the combination of caloric restriction and appropriate exercise in aging individuals (Fig. 7B). In recent years, certain anti-aging drugs, such as Nicotinamide mononucleotide (NMN), have been suggested as potential agents to retard aging by enhancing tissue functions [37, 38]. Employing western blot and RT-qPCR methods, we investigated the expression of HNRNPC in the adipose tissue of aging mice after NMN supplementation. The results indicated a significant increase in HNRNPC levels following NMN treatment (Fig. 7C-D). Furthermore, the expression of LCP1 was significantly increased in response to dietary changes, exercise, or NMN treatment (Fig. 7E-H). These results suggest that the upregulation of HNRNPC and LCP1 levels might mediate the process of metabolic improvement that delays the aging progression.

## DISCUSSION

Recently, several studies have shown that m^6^A modification is widely present in aging tissues, and m^6^A regulators have different roles in aging and related diseases (38). Interestingly, the same m^6^A-regulated genes can have contradictory functions in different aging-related diseases, while other m^6^A-regulated genes can promote or inhibit aging within the same disease context (17, 18, 39, 40). Additionally, the regulation of aging by m^6^A exhibits temporal and spatial characteristics. Different tissues of the same aging individual, such as the liver, heart, and muscle tissues, and even different regions of the same tissue, have varying levels of m^6^A (41, 42). These studies underscore the coplex nature of the involvement of m^6^A in aging, necessitating tissue-specific analyses. In this study, we found that the overall m^6^A level of adipose tissue of aged mice was decreased, and the differential expression of HNRNPC was the most significant m^6^A regulatory factor. We do not deny that the methyltransferases and desmethyltransferases of m^6^A may play a role in the process of adipose tissue aging, and the results of our datasets revealed that the expression of these regulators varies. Although the metabolic profiles of *Hnrnpc-APKO* mice looked like aging, the m^6^A levels found in the adipose tissue of *Hnrnpc-APKO* mice were the same as the control mice (Fig. S5A). Furthermore, we confirmed *in vitro* and *in vivo* that HNRNPC regulated the adipogenesis capacity of adipose tissue. These data suggested that HNRNPC played an essential role in adipogenesis decay in aging despite m^6^A changing. This study focuses on the effects of HNRNPC as an m^6^A reader on adipose tissue aging, but the reasons for decreased m^6^A levels in aging adipose tissue warrant further investigation.

Several studies have demonstrated that the m^6^A regulators participate in adipogenesis, for example, FTO and ALKBH5-mediated demethylation are involved in adipogenic differentiation of 3T3-L1 or MSC [14, 20, 39]. But some reports showed that the regulation of adipogenesis by m^6^A is inconsistent. Yao et al. found that Silencing of METTL3 inhibited adipogenesis by decreasing mRNA m^6^A levels of Janus kinase 1 (*Jak1*) and alleviated YTHDF2-dependent *Jak1* mRNA degradation in porcine bone marrow-derived MSCs (BMSC) [40]. However, another research showed that METTL3 overexpression increased m^6^A levels and inhibited adipogenesis in porcine preadipocytes [41]. These findings indicate that the regulation of adipogenesis by m^6^A may be tissue-source specific. By regulating transcriptional cascade and extracellular signals, m^6^A readers also play a critical role in adipogenesis. YTHDF2 is involved in adipogenesis by regulating the mitotic clonal expansion (MCE) process during 3T3-L1 adipocyte differentiation [42, 43]. YTHDF2 also prevented adipogenesis by modulating the JAK/STAT signaling pathway in bone marrow stem cells, porcine preadipocytes, and 3T3-L1 [40, 44]. In pigs, YTHDF1 was involved in the adipogenesis of the intramuscular fat [45, 46]. Our data shows that HNRNPC promotes adipogenesis by regulating cytoskeletal remodeling.

Adipocyte formation is accompanied by cytoskeletal remodeling. Previous studies have shown that after MSC contacting inhibition, F-actin shows disruption by treatment with lipogenic inducers, and cells in this state are more prone to lipogenesis than osteogenesis [32]. However, one study showed that adipogenesis is inhibited by knocking down the zinc finger CCCH-type containing 10 (*Zc3h10*) MSC accompanied by disrupted cytoskeleton at the early stage of differentiation into adipocytes [47]. These studies suggest that the changes in the cytoskeleton before and after inducing differentiation are unique. In our study, we discovered that the skeleton-related gene LCP1 was the downstream target gene of HNRNPC, and HNRNPC regulated LCP1 through the binding to the m^6^A motif of *Lcp1*. We further verified that LCP1 overexpression could reverse the cytoskeletal disruption and attenuated adipogenesis due to HNRNPC knockdown.

LCP1 is involved in the cell differentiation process [48]. It reinforces the cytoskeleton structure by cross-linking with F-actin filaments and works as a scaffold for signaling pathways involved in B-cell biological processes [49]. Furthermore, studies have shown that LCP1 plays a role in the assembly of nascent seals (NSZs) in the early stages of osteoclast seal ring formation [50]. It has also been found that low LCP1 expression inhibits lipid accumulation in 3T3-L1 cells, but the specific mechanism is yet to be elucidated [51]. Our findings suggested that LCP1 can promote adipogenesis by remodeling the cytoskeleton, which is consistent with previous results.

It has been shown that the cytoskeletal component F-actin shows different age-related alterations in mouse support cells, which is considered to be a marker of testicular senescence [52]. In our study, we observed the cytoskeletal disruption in etoposide-treated SVF (Supplementary Fig. 5B-E), which was similar to the cytoskeletal state that occurred after HNRNPC knockdown. Our findings further validate that HNRNPC may be used as a marker to evaluate the aging process of adipose tissue.

The aging process can be mitigated through a sensible diet, appropriate calorie restriction, and a combination of exercise. Additionally, certain anti-aging drugs have shown a significant capacity to enhance the function of various tissues in the aging organism, effectively slowing down the aging process [34-38, 53]. Our study demonstrated that the expression of HNRNPC and its downstream LCP1 was upregulated following these remedial measures, providing further support for considering HNRNPC as a potential target for anti-aging interventions.

## Conclusion

This study discovered that the reduced expression of m^6^A reader HNRNPC suppresses adipogenesis during aging, leading to insulin resistance, elevated expression of aging-related and inflammation-related genes, reduced lipogenesis-related genes, and decreased energy metabolism. HNRNPC level could be upregulated following the anti-aging interventions. Therefore, HNRNPC could be a new potential target to combat adipose tissue aging and related metabolic diseases.

## Supplementary Materials

The Supplementary data can be found online at: www.aginganddisease.org/EN/10.14336/AD.2024.0132.



## Data Availability

Data in support of the results of this study are available on appropriate request from the corresponding author.
