# Supplementary material for: m6A Reader HNRNPC Facilitates Adipogenesis by Regulating Cytoskeletal Remodeling through Enhanced Lcp1 mRNA Stability
Source: Aging Dis. 2024 May 14;16(2):1080–98. doi: 10.14336/AD.2024.0132 (PMC11964444; doi:10.14336/AD.2024.0132)
Supplement: Supplementary file 1 [file AD-16-2-1080-s.pdf]

SUPPLEMENTARY DATA

m^6A Reader HNRNPC Facilitates Adipogenesis by Regulating Cytoskeletal Remodeling through Enhanced *Lcp1* mRNA Stability

Wenhua Xie, Yewei Cui, Lingzhi Yue, Ting Zhang, Chenglong Huang, Xinyu Yu, Dan Ma, Dongfang Liu, Rui Cheng, Xueya Zhao, Xi Li

© 2024. Xie W et al. Published online at http://www.aginganddisease.org/EN/10.14336/AD.2024.0132

SUPPLEMENTARY DATA

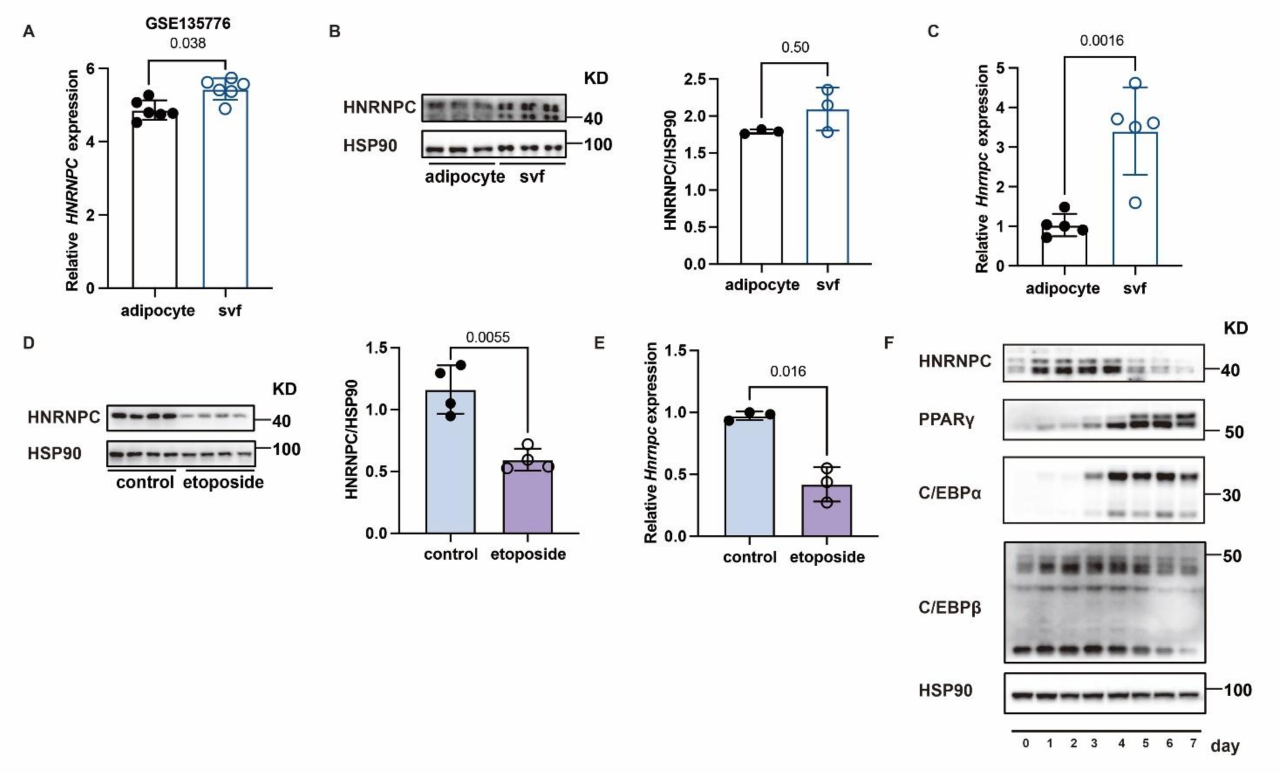

Supplementary Figure 1. The levels of m^{6}A regulators and HNRNPC in adipose tissues during aging. A. The mRNA expression of *HNRNPC* in the adipocyte (n = 6) and SVF (n = 6) of humans in GSE135776. **B.** The protein of HNRNPC in the adipocyte (n = 3) and SVF (n = 3). **C.** The mRNA expression of *Hnrnpc* in the adipocyte (n = 5) and SVF (n = 5) of mice. **D.** Western blot analysis of HNRNPC in control (n = 4) and etoposide-treated SVF (n = 4). **E.** RT-qPCR analysis of *Hnrnpc* in control (n = 3) and etoposide-treated SVF (n = 3). **F.** Expression of C/EBPβ, C/EBPα, PPARγ and HNRNPC in 3T3-L1 cells induced differentiation into mature adipocytes at different time points. All data were shown as mean ± SD. After performing the Shapiro-Wilk normality test to examine the normal distribution, a paired two-tailed Student′s t-test or unpaired two-tailed Student′s t-test with Welch’s correction was utilized to assess the significance between the two groups. $P < 0.05$ was considered statistically significant.

© 2024. Xie W et al. Published online at http://www.aginganddisease.org/EN/10.14336/AD.2024.0132

SUPPLEMENTARY DATA

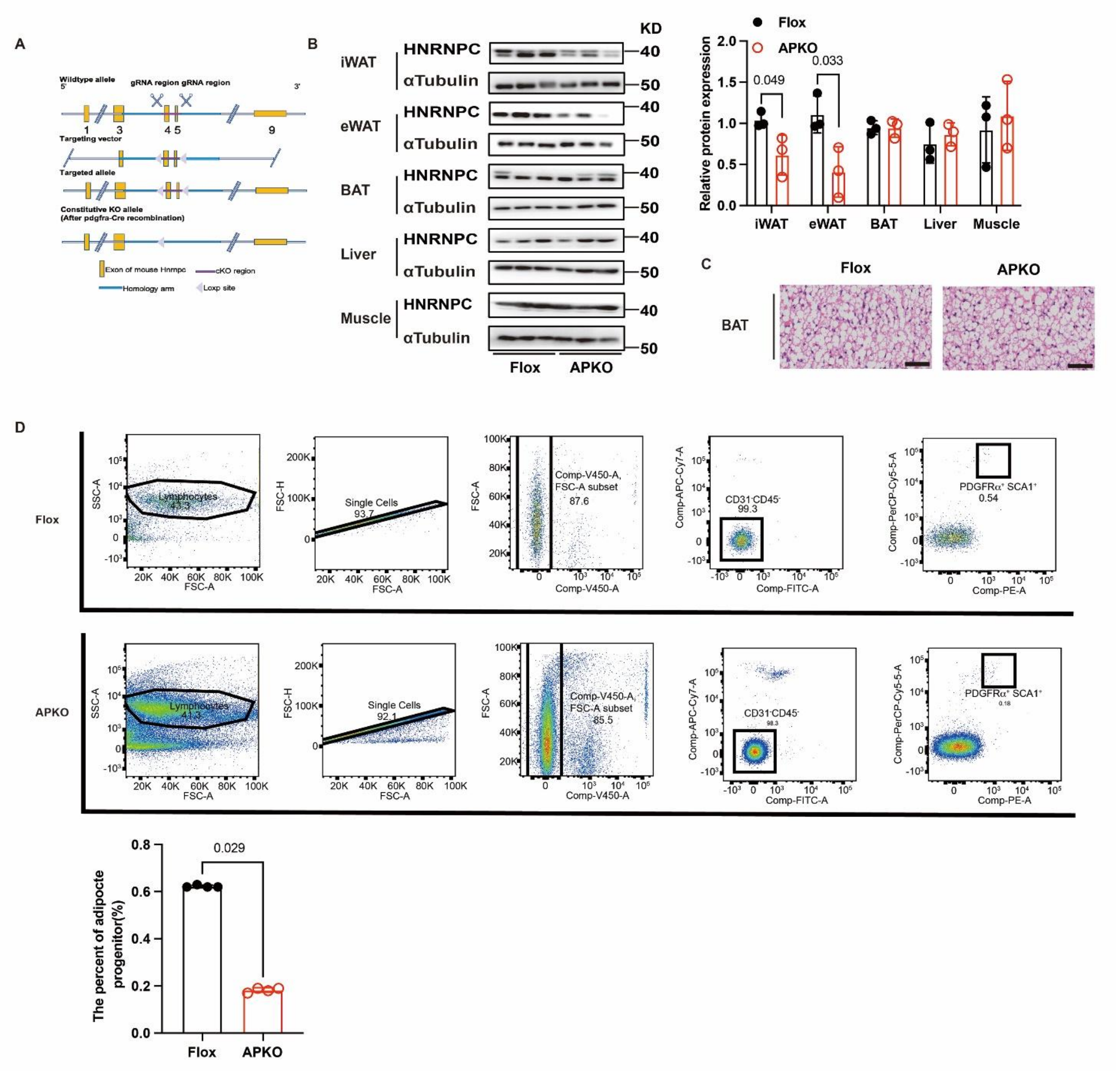

Supplementary Figure 2. Knocking down HNRNPC inhibits adipogenesis. A. The construction diagram of Flox and APKO mice. **B.** The protein expression of HNRNPC in Flox and APKO mice (iWAT (n = 3) eWAT (n = 3) BAT (n = 3) Liver (n = 3) and Muscle (n = 3)). **C.** The histological morphology of BAT in Flox (n = 4) and APKO (n = 4) mice. **D.** The percentage of adipocyte progenitor cells which were labeled $CD31^-CD45^-Pdgfra^+Sca1^+$ antibody in Flox (n = 4) and APKO (n = 4) mice. All data were shown as mean ± SD. After performing the Shapiro-Wilk normality test to examine the normal distribution, the unpaired, two-tailed Student's t-test with Welch’s correction was utilized to assess the significance between the two groups. $P < 0.05$ was considered statistically significant.

© 2024. Xie W et al. Published online at http://www.aginganddisease.org/EN/10.14336/AD.2024.0132

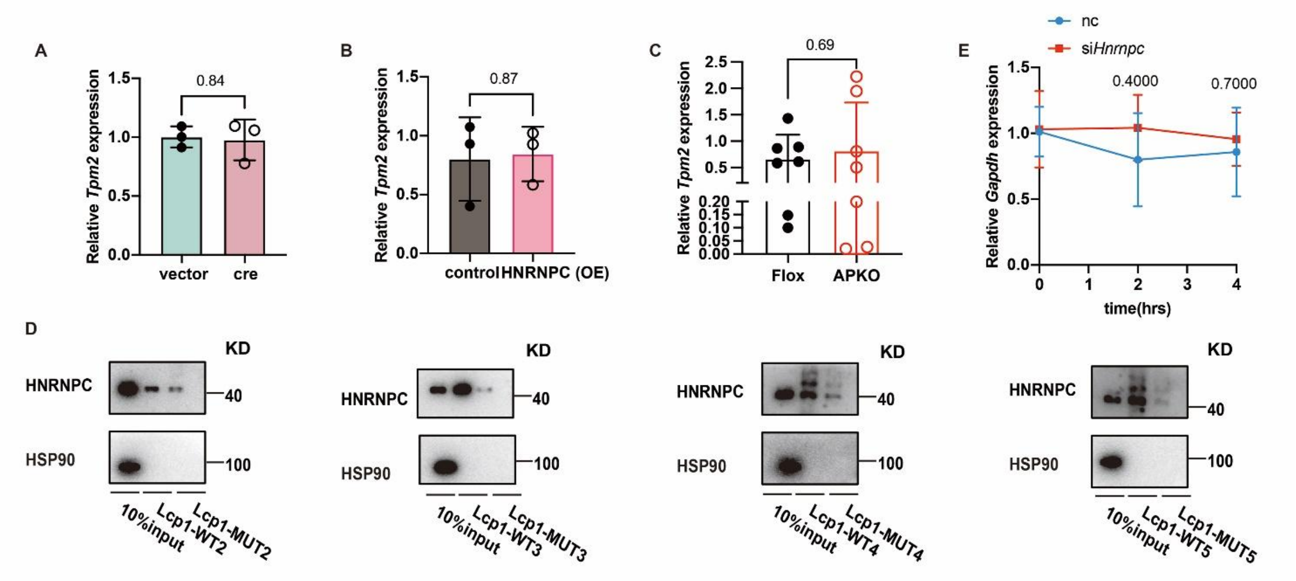

Supplementary Figure 3. HNRNPC regulates *Lcp1* mRNA stability through binding to the m^{6}A motif of *Lcp1*. A-C. The mRNA expression of *Tpm2* in HNRNPC deficient and overexpression HNRNPC. **D.** The western blot of HNRNPC in RNA-Pull down. **E.** The expression of *Gapdh* in HNRNPC deficiency with actinomycin D, nc (n = 3), si*Hnrnpc* (n = 3). All data were shown as mean ± SD. After performing the Shapiro-Wilk normality test to examine the normal distribution, an unpaired two-tailed Student′s t-test or unpaired two-tailed Student′s t-test with Welch’s correction was utilized to assess the significance between the two groups. P < 0.05 was considered statistically significant.

© 2024. Xie W et al. Published online at http://www.aginganddisease.org/EN/10.14336/AD.2024.0132

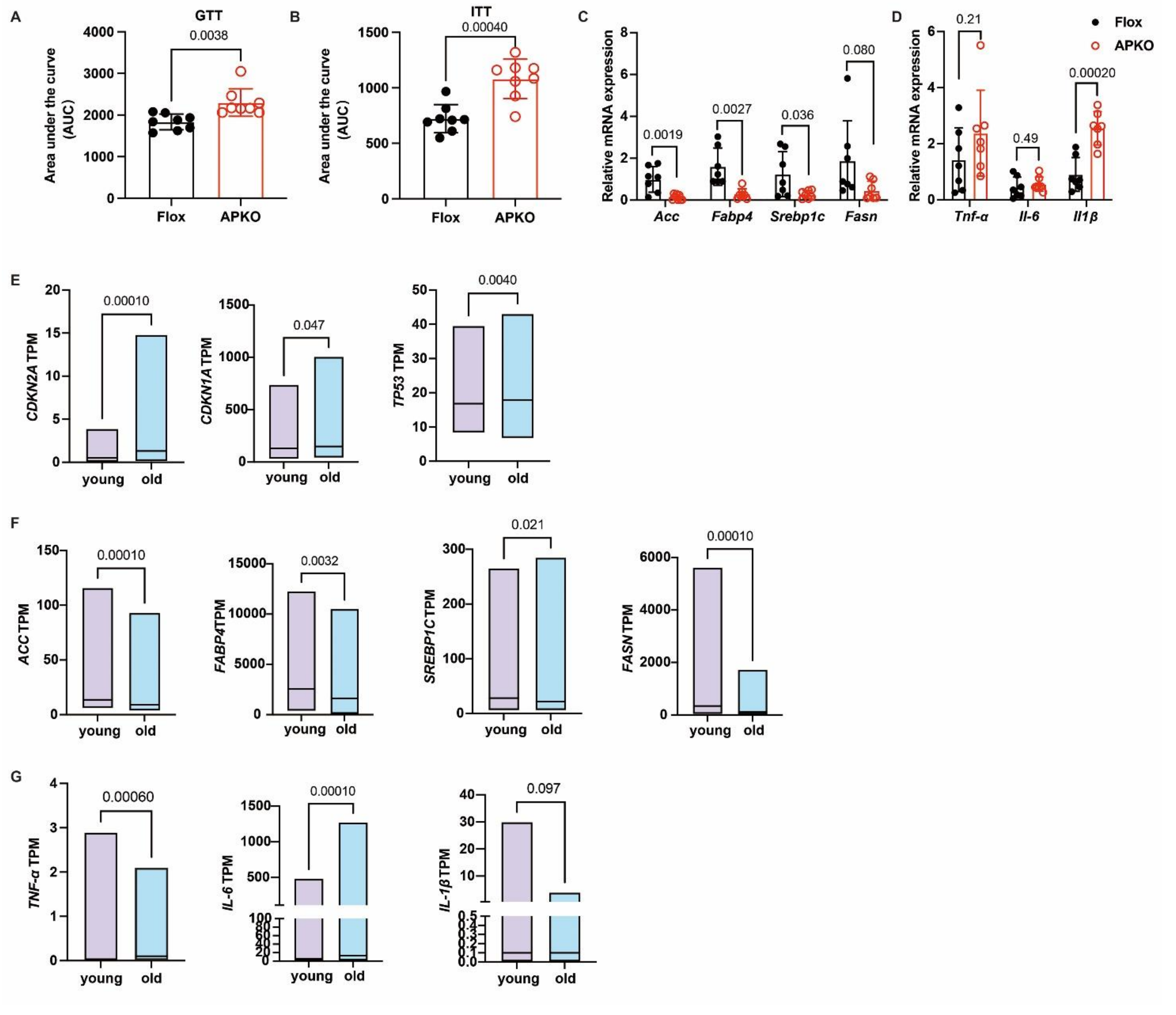

Supplementary Figure 4. The area under the curve of GTT and ITT, the aging markers in human adipose tissue. A-B. The area under the curve (AUC) of GTT and ITT in Flox (n=8) and APKO (n=8) mice. **C-D**. RT-qPCR analysis of *Acc, Fabp4, Srebp1c, Fasn, Tnf-a, Il-6 and Il-1b* in Flox (n=7) and APKO (n=7) mice. **E-G.** The expression of *CDKN2A, CDKN1A, TP53, ACC, FABP4, SREBP1C, FASN, TNF-a, IL-6 and IL-1b* in young (n=110) and old (n=237) human subcutaneous adipose tissue. All data were shown as mean ± SD. After performing the Shapiro-Wilk normality test to examine the normal distribution, an unpaired two-tailed Student′s t-test or non-parametric Mann-Whitney test was utilized to assess the significance between the two groups. P < 0.05 was considered statistically significant.

© 2024. Xie W et al. Published online at http://www.aginganddisease.org/EN/10.14336/AD.2024.0132

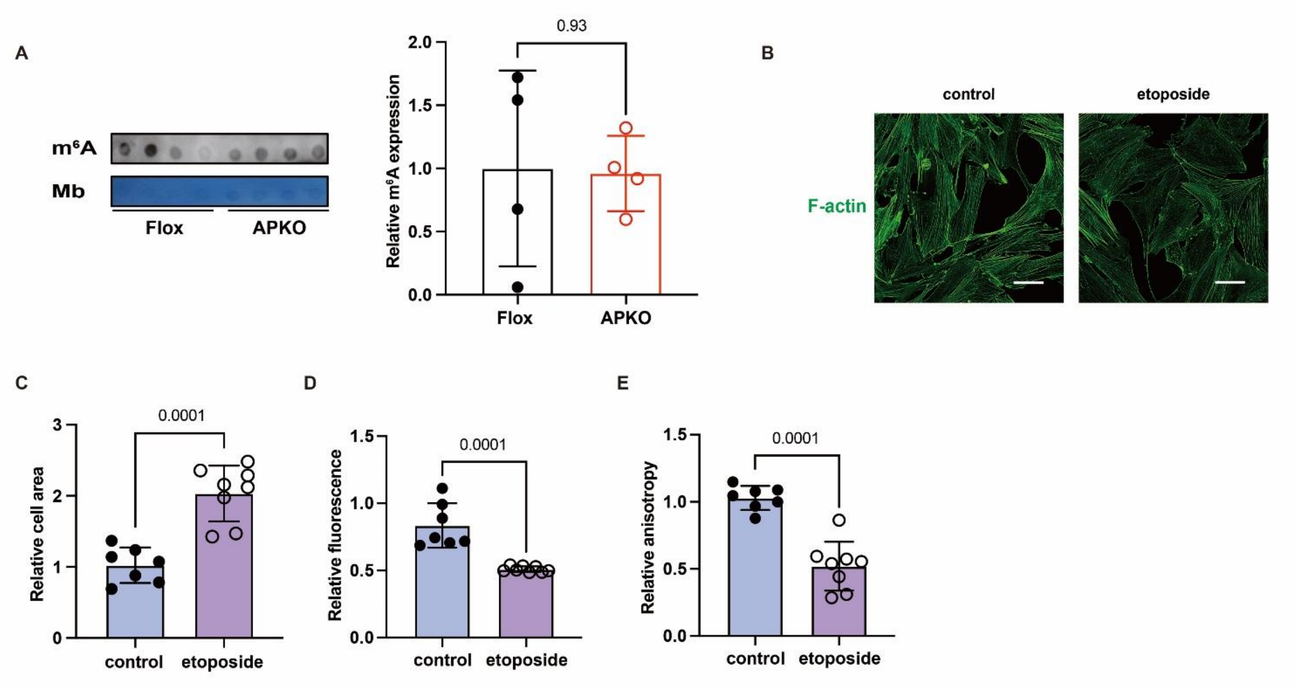

Supplementary Figure 5. The F-actin in the aging cell. A. The level of m^{6}A modification in Flox (n = 4) and APKO (n = 4) mice. **B.** The F-actin fluorescence confocal. **C–E.** The relative cell area, the relative fluorescence, and the relative anisotropy in the control group (n = 7) and etoposide-treated group (n = 8). Scale bars, 50 μm. All data were shown as mean ± SD. After performing the Shapiro-Wilk normality test to examine the normal distribution, an unpaired two-tailed Student′s t-test or unpaired two-tailed Student′s t-test with Welch's correction was utilized to assess the significance between the two groups. $P < 0.05$ was considered statistically significant.

© 2024. Xie W et al. Published online at http://www.aginganddisease.org/EN/10.14336/AD.2024.0132
